# Virus Host Jumping Can Be Boosted by Adaptation to a Bridge Plant Species

**DOI:** 10.3390/microorganisms9040805

**Published:** 2021-04-11

**Authors:** Sandra Martínez-Turiño, María Calvo, Leonor Cecilia Bedoya, Mingmin Zhao, Juan Antonio García

**Affiliations:** Department of Plant Molecular Genetics, Centro Nacional de Biotecnología (CNB-CSIC), Campus Universidad Autónoma de Madrid, Darwin 3, 28049 Madrid, Spain; mariacalvo83@gmail.com (M.C.); leonorbedoya@gmail.com (L.C.B.); mingminzh@163.com (M.Z.)

**Keywords:** host jumping, viral evolution, trade-off, plant virus, RNA virus, potyvirus, *Plum pox virus*, VPg, eIF4E

## Abstract

Understanding biological mechanisms that regulate emergence of viral diseases, in particular those events engaging cross-species pathogens spillover, is becoming increasingly important in virology. Species barrier jumping has been extensively studied in animal viruses, and the critical role of a suitable intermediate host in animal viruses-generated human pandemics is highly topical. However, studies on host jumping involving plant viruses have been focused on shifting intra-species, leaving aside the putative role of “bridge hosts” in facilitating interspecies crossing. Here, we take advantage of several VPg mutants, derived from a chimeric construct of the potyvirus *Plum pox virus* (PPV), analyzing its differential behaviour in three herbaceous species. Our results showed that two VPg mutations in a *Nicotiana clevelandii*-adapted virus, emerged during adaptation to the bridge-host *Arabidopsis thaliana*, drastically prompted partial adaptation to *Chenopodium foetidum*. Although both changes are expected to facilitate productive interactions with eIF(iso)4E, polymorphims detected in PPV VPg and the three eIF(iso)4E studied, extrapolated to a recent VPg:eIF4E structural model, suggested that two adaptation ways can be operating. Remarkably, we found that VPg mutations driving host-range expansion in two non-related species, not only are not associated with cost trade-off constraints in the original host, but also improve fitness on it.

## 1. Introduction

Emerging viral diseases are frequently the result of host jumps, when a pathogen gains the ability to infect a new species [1,2]. Host jumping has received particular attention in the case of animal and human diseases, with the host range breadth being a major determinant of bacterial but also viral emerging outbreaks [3,4,5]. Interspecies jumping is not uncommon among plant viruses, as evidenced by the fairly diverse host ranges and large host range width disparities in viruses derived from a recent radiative evolution [6], as well as by the frequent inconsistencies observed between the pathogen and host phylogenies [7]. Indeed, the host range expansion is considered pivotal for emergence of plant pathogens, especially plant viruses [8,9]—a phenomenon frequently linked to epidemic outbreaks in crops causing substantial yield losses [10,11]. Viral host jumping goes hand in hand with the concept of adaptive trade-off, according to which a pathogen cannot simultaneously maximize its fitness in all hosts. Thus, viral adaptation to a particular species normally implies a fitness cost in alternative species, and generalist viruses infecting numerous hosts evolve to reach fitness values maximized among hosts, but lower than the optimum they would have reached if had adapted to a single host [12].

The genus *Potyvirus* (family *Potyviridae*), is one of the most important groups of plant viruses [13]. The genome of potyviruses is a positive-sense single-stranded RNA of ~10 kb, whose 5′ end remains attached to the viral genome-linked protein (VPg) [14]. The potyviral genome is translated into a large polyprotein that is proteolytically processed to render at least 9 final products [15]. Moreover, frameshifts resulting from RNA polymerase slippage allow production of additional transframe products [16,17,18].

The intrinsic disorder that characterizes VPg protein [19,20,21] enables it to play multiple functions during viral infection [22,23,24]. VPg is involved in viral RNA translation probably both by recruiting host factors to promote translation initiation and by nucleating a protein complex around the viral RNA that protects it from the RNA silencing mechanism and facilitates its traffic to polysomes [25]. The interaction of VPg with the eukaryotic translation initiation factor eIF4E and/or its isoform eIF(iso)4E plays an important role in these functions. Compatibility/incompatibility of this interaction is a typical example of host-pathogen coevolution, according to which the virus evolves to match the host factor through adapting VPg, favoured by the large mutational robustness conferred by the intrinsic disorder of this protein [26], and the plant evolves to avoid such interaction [27,28]. Indeed, the lack of a functional interaction between VPg and eIF4E or eIF(iso)4E causes most cases of recessive resistance to potyviruses [29,30]. However, VPg-eIF4E/(iso)4E appears to be only one component of a more complex network involving multiple potyviral components and host factors [31]. This assumption is supported by the identification of HCPro as another interaction partner of eIF4E and eIF(iso)4E [32] and by the fact that sometimes breakdown of eIF4E-mediated anti-potyvirus resistance has been found associated with mutations in proteins P1 [33], P3 [34] and CI [35,36].

The potyvirus *Plum pox virus* (PPV) is the most serious viral agent of stone fruits, which causes sharka, a devastating disease affecting *Prunus* species [37]. In nature, PPV infects most of *Prunus* species, but it can also infect a wide range of herbaceous plants under experimental conditions [38,39]. It has been described up to ten different PPV strains, quite distinct in terms of their host range [40,41]. Among them, PPV-Dideron (D) is the most widely distributed PPV strain, infecting numerous *Prunus* species, while PPV-Cherry (C) is characterised by a restricted natural host range, limited to cherry trees [42,43]. Isolates of these strains also have remarkable differences on infectivity in experimental herbaceous hosts. SwCMp and Rankovic (R) isolates, respectively belonging to PPV-C and PPV-D strains, are highly infectious in *Nicotiana clevelandii* and *Nicotiana benthamiana*; however, while the isolate PPV-R (D strain) causes local lesions in *Chenopodium foetidum* and systemic infections in *Arabidopsis thaliana*, PPV-SwCMp (C strain) is not able to infect these two plant species [39,44]. Using chimeric viral cDNA clones, nuclear inclusion a (NIa), which includes VPg and a protease domain, was identified as the major pathogenicity determinant preventing PPV-SwCMp infection in *A. thaliana* and *C. foetidum* [44]. In the course of the same work, Calvo et al. identified specific mutations at the VPg protein of PPV-SwCMp, arisen as result of adaptation in *A. thaliana* of a R/SwCMp PPV chimera, which were suggested would contribute to gain infectivity in this host by enabling compatible interactions between VPg and eIF(iso)4E [44].

In this work, we have confirmed that the VPg mutations detected in the viral progeny of *A. thaliana* facilitate the efficient infection of this host. Likewise, we have shown that these mutations also provide an infectivity gain in a second restrictive host, *C. foetidum*, without any trade-off in the original host *N. clevelandii*. Furthermore, we discuss the possible relevance of the mutated residues in the viral protein for VPg/eIF(iso)4E compatibility and virus host range definition. The possibility that wild plants serve as bridges that facilitate “host jumps” and emergence of new diseases is also addressed.

## 2. Materials and Methods

### 2.1. Viral cDNA Clones

Three previously obtained PPV full-length cDNA clones were employed. pICPPV-NK-lGFP [45] and pICPPV-SwCM [46], respectively derive from the Nicotiana-adapted PPV isolates, PPV-R, belonging to strain D, and PPV-SwCMp from strain C. The chimeric clone pICPPV-VPgSwCM-R carries the VPg sequence from PPV-SwCMp into the PPV-R backbone [44].

Effect of point mutations in the VPg sequence was assayed using three constructs ad-hoc obtained. Amino acids substitutions P114S or F163L in the VPg protein sequence were engineered into the chimeric clone pICPPV-VPgSwCM-R, by replacing the nucleotide triplet CCA by TCA at position 1968–1970 (giving rise to the construct P114S) or substituting the nucleotide triplet TTC by CTC at position 2017–2019 (construct F163L). The double mutant carrying both P114S and F163L substitutions (P114S-F163L) was also generated (Supplementary Figure S1). These point mutations were introduced by using the three-step PCR-based mutagenesis method [47], using the mutators and flanking primers listed in Supplementary Table S1. First mutagenic PCR reactions used the plasmid pICPPV-VPgSwCM-R as template; then, products of these reactions were mixed and employed as templates for a second round PCR. Final overlapping amplicons, digested with *Xho*I (partially in the case of F163L mutagenesis) and *Nru*I, served to replace the corresponding fragment from pICPPV-VPgSwCM-R. Double mutant P114S-F163L was obtained following the same strategy, but by using as template the previously obtained P114S construct (Supplementary Figure S1 and Supplementary Table S1).

### 2.2. Viral Inoculation and Plants Growth Conditions

For mechanical hand inoculation, approximately 15 µL of leaf extracts or plasmid DNAs, (1.0–1.5 μg/μL), were distributed on three leaves of young plants of *N. clevelandii, C. foetidum* or *A. thaliana* (four- to six-leaf stage), previously dusted with Carborundum powder. Leaf extracts used as inocula were obtained as previously described [44] from *N. clevelandii* or *A. thaliana* leaves already infected. Primary inoculation of *A. thaliana* plants was done by bombardment with microgold particles coated with DNA using a Helios gene gun (Bio-Rad, Hercules, CA, USA) [48]. Microcarrier cartridges were prepared with 1.0 μm diameter gold particles coated at a DNA loading ratio of 2 μg/mg gold and a microcarrier loading amount of 0.5 mg/shot. One cartridge, shot twice onto two leaves of each plant, under a helium pressure of 7.0 bar, was employed.

All plants were grown under glasshouse conditions with 16 h of light photoperiod using natural and supplementary illumination, at a temperature range of 19–23 °C and 67–70% relative humidity. *A. thaliana* ecotype Columbia (Col-0) seeds were vernalized at 4 °C and in vitro grown on MS medium (Sigma Aldrich, St. Louis, MO, USA) containing 0.5 % (*w/v*) of sucrose and 1% (*w/v*) of agar. Once germinated, seedlings were kept for two weeks in a phytotron (Neurtek, Eibar, Spain) under a 14 h photoperiod, at 22 °C and 50–60% relative humidity. After planted out to soil-vermiculite (3:1), plants were grown in controlled environment chambers as mentioned above.

### 2.3. Assessment of Viral Infection

Viral infection was monitored by visual inspection of PPV-induced symptoms and by immunoblot analysis, as described by Calvo et al. [44]. Infection in *C. foetidum* plants was evaluated by registering over time the total number and type of local lesions, discriminating between doubtful, chlorotic or necrotic lesions of variable intensity.

### 2.4. Viral Progeny Characterization and Sequence and Structure Analyses

For characterization of the viral progeny, appropriate viral DNA fragments covering the entire VPg sequence were amplified from systemically infected tissue of *A. thaliana* and *N. clevelandii,* by immune-capture-RT-PCR (IC-RT-PCR), as previously described [44]. Alternatively, viral progeny in *C. foetidum* was analyzed by direct RT-PCR from total RNA obtained from individual lesions or entire leaves, employing the FavorPrep Plant Total RNA Purification Mini-Kit (Favorgen Biotech, Ping-Tung, Taiwan). Amplification of a region containing the VPg sequence was done using oligos 2295 and 2277, after inoculation with pICPPV-VPgSwCM-R-derived plasmids or with their viral progenies, or with oligos SM16-F and SM17-R after inoculation with pICPPV-NK-lGFP or its viral progeny (Supplementary Table S1). Sanger sequencing of amplified fragments was performed by Macrogen Europe (Amsterdam, The Netherlands) using primers SM18-F and/or SM19-R.

For the identification of the *C. foetidum* eIF(iso)4E [Cf-eIF(iso)4E] sequence, total RNA was extracted from *C. foetidum* leaves, as mentioned above, and cDNA was synthetized from it by using the Invitrogen SuperScript III Reverse Transcriptase and Invitrogen hexameric random primers (both from Thermo Fisher Scientific, Rockford, IL, USA), following manufacturer instructions. From the cDNA product, treated with RNase H, a Cf-eIF(iso)4E gene fragment was amplified by using the Thermo Scientific Phusion High-Fidelity DNA Polymerase (Thermo Fisher Scientific, Rockford, IL, USA) and a pair of degenerate oligonucleotides, SM110-F-deg and SM111-R-deg (Supplementary Table S1), designed on the basis of the known-sequences of *Chenopodium quinoa* eIF(iso)4E (LOC110697254 and LOC110692931). Sanger sequencing of the amplified gene fragment was performed by Macrogen Europe (Amsterdam, The Netherlands) using the same primers employed for amplification (Supplementary Table S1).

Alignment of multiple protein sequences was carried out with the Clustal Omega web server [49] (www.ebi.ac.uk/Tools/msa/clustalo, accessed on 1 April 2021) using default parameters. Sequences of *Potato virus Y* (PVY) VPg (VPg PVY), human eIF4E (h-eIF4E) and other plant eIF(iso)4E proteins, were retrieved from the Protein Data Bank (PDB, www.rcsb.org/pdb/ accessed on 1 April 2021) [50].

Putative spatial localization of specific residues relevant for this study, were mapped over a HADDOCK-derived model, complexing h-eIF4E and PVY VPg, previously generated by Countinho de Oliveira et al. [51]. Equivalences between residues of the model and eIF(iso)4Es/PPV VPg were obtained on the basis of corresponding protein alignments. 3D protein structures were visualized by using PyMOL Molecular Graphics System, version 2.1.1 (Schrödinger).

## 3. Results

### 3.1. Point Mutations at the VPg Protein in an Avirulent Chimeric Construct of PPV Promote Infection of Arabidopsis thaliana

**Figure 1:**
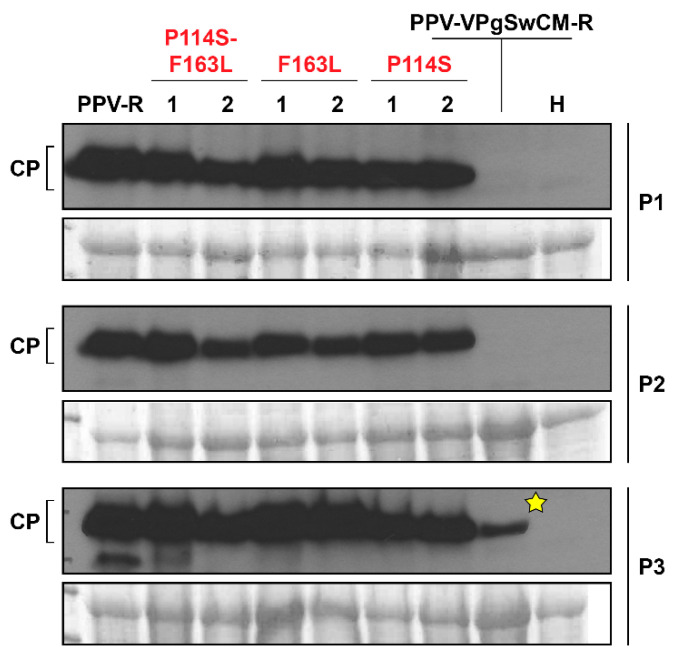
Effect of VPg mutations on *A. thaliana* infection by *Plum pox virus* (PPV). *A. thaliana* plants were inoculated by biolistic with the chimeric clone pICPPV-VPgSwCM-R, its indicated mutant variants (two independent clones, 1 and 2), or the PPV-R clone pICPPV-NK-lGFP. Extracts from upper non-inoculated leaves collected at 15 days after inoculation were subjected to CP-specific immunoblot analysis. Three individual plants (P1, P2 and P3), inoculated with the specified viruses, were analyzed. An extract of healthy plants (H) was used as a negative control. Blots stained with *Ponceau* red showing the large subunit of the ribulose-1,5-bisphosphate carboxylase-oxygenase (RuBisCO) are included as loading controls. The yellow star indicates that the progeny virus of this plant had incorporated a mutation in the VPg sequence (P114S).

Previous works had shown that, while the PPV-R isolate belonging to the D strain, efficiently infects *A. thaliana*, a chimeric construct bearing the VPg sequence from a PPV isolate belonging to the C strain in the backbone of PPV-R (PPV-VPgSwCM-R) rarely infects this host [44]. Infection of *Arabidopsis* by PPV-VPgSwCM-R appeared to be promoted by emergence of point mutations at the SwCMp VPg coding sequence [44]. In order to examine whether the detected changes at VPg sequence are solely responsible for the gain of infectivity, we assayed the effect of these mutations, proline to serine at position 114 (P114S) and phenylalanine to serine at position 163 (F163L), separately engineered into the cDNA clone of the PPV-VPgSwCM-R chimera. Besides, although these modifications had not been concomitantly detected in *A. thaliana*, both changes were also introduced together into the chimeric clone (Supplementary Figure S1). The mutated constructs and appropriate controls were biolistically inoculated in *A. thaliana* plants (Supplementary Figure S2A). Viral accumulation was checked by an immunoblot assay at 15 dpi (Figure 1). VPg mutants P114S and F163L showed similar viral CP accumulation as the PPV-R positive control. RT-PCR amplification and sequencing of the complete VPg gene showed no sequence changes in the viral progeny of the three plants infected with each tested mutant. These results confirm previous assumption launched by Calvo et al. [44] that both P114S and F163L mutations were able, by themselves, to facilitate adaptation of the PPV-VPgSwCM-R chimera to *Arabidopsis*. As expected, the wild type PPV-VPgSwCM-R chimera hardly accumulates in inoculated plants, being undetectable in two of the three plants tested. A weak CP signal was detected in the third analyzed plant; however, the analysis of its viral progeny revealed that a P114S mutation had been introduced in the VPg coding sequence, further confirming the relevance of this change for adaptation to *Arabidopsis*.

The double mutant P114S-F163L was also infectious and genetically stable in *A. thaliana* plants (Figure 1). Although the existence of certain differences at early times of infection cannot be excluded, all VPg mutations seem to allow similar viral accumulation, comparable with that of the positive control PPV-R (Figure 1).

**Figure 2:**
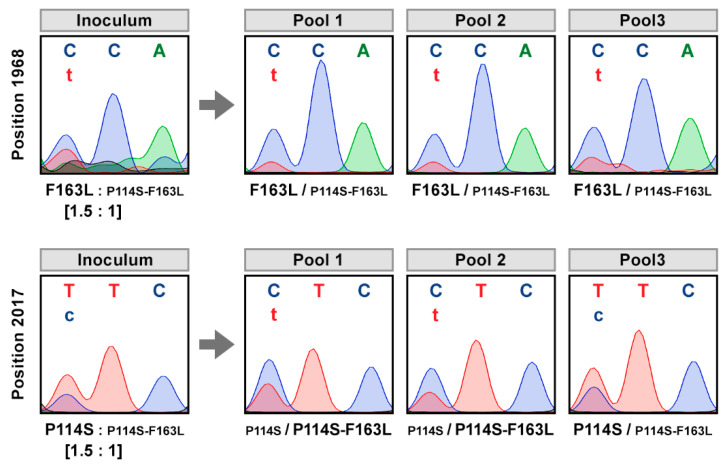
Sequence analysis of viral progeny from *Arabidopsis thaliana* exposed to mixed infections with competing viruses. DNAs of pICPPV-VPgSwCM-R chimeric clones modified by the specified mutations were mixed at the indicated ratio and biolistically inoculated into six *A. thaliana* plants. Viral progenies were analyzed in pools of two plants by reverse transcription-polymerase chain reaction (RT-PCR), then sequencing a cDNA fragment covering the VPg coding sequence. Images show the chromatograms of VPg codons 114 (position 1968–1970 in the viral genome) or 163 (position 2017–2019 in the viral genome). Identified viruses are indicated beneath the chromatograms; smaller letters indicate lower accumulation. A similar result was obtained in a replicate assay.

To find out whether the two specific mutations in the VPg could have a synergist contribution to the adaptation to the new host, appropriate competition assays were carried out. Mixtures of cDNAs, each corresponding to the double mutant and one of the single mutants, were biolistically inoculated in *A. thaliana* plants, at concentrations adjusted to achieve a 1.5:1 ratio, thus conferring some advantage in favour to individual mutations (Supplementary Figure S2B). Systemic infection was monitored by immunodetection of CP in upper non-inoculated leaves at 21 dpi. Genotyping of viral progenies was carried out by IC-RT-PCR amplification and sequencing of the VPg coding sequence from three pools of two plants infected with each mutant combination. Examination of viral progeny after the competition F163L vs P114S-F163L did not reveal important differences in the fitness of any of the two types of viruses, which coexisted in all three analyzed pools of plants, maintaining the differences between them already existing in the inoculum (Figure 2). Thus, the change P114S does not seem to provide any competitive advantage when it is together with the mutation F163L. Similarly, when mutants P114S-F163L and P114S competed, both viruses coexisted in the three pools of analyzed plants. However, the ratio of the DNA inoculum was reversed in two of the analyzed pools, and the double mutant, despite its lesser initial representation, became the majority virus (Figure 2). These results suggest that the enhancement of viral fitness in *A. thaliana* conferred by the F163L mutation in the VPg of PPV-SwCMp might be greater than that provided by the P114S mutation.

### 3.2. PPV Adaptation to A. thaliana, via Specific Mutations in VPg, Does Not Have a Fitness Cost in Nicotiana clevelandii

Changes introduced in the chimeric virus PPV-VPgSwCM-R that facilitate its amplification in *A. thaliana* are expected to be associated with a loss of fitness in *N. clevelandii*, a host in which the non-mutated virus is completely adapted. To evaluate this possibility, four *N. clevelandii* plants were manually inoculated by hand rubbing with DNAs of the two single mutants, P114S or F163L, as well as with that of the double mutant P114S-F163L. Both, PPV-R and the non-mutated chimera, PPV-VPgSwCM-R, were included as positive controls of infection (Supplementary Figure S2A). The results showed that the three viruses with mutations in VPg systemically infected *N. clevelandii* with comparable efficiency. Overall, the onset and severity of disease symptoms (not shown), as well as levels of viral CP accumulation, were similar for all the three viruses and indistinguishable from those induced by the positive controls (Supplementary Figure S3).

**Figure 3:**
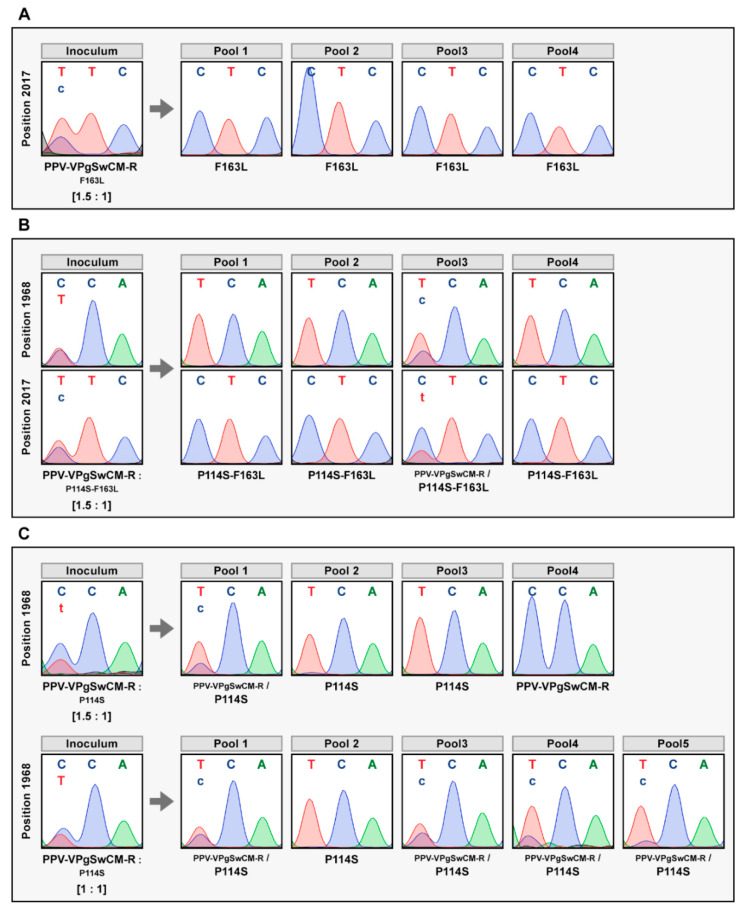
Sequence analysis of viral progeny from *Nicotiana clevelandii* exposed to mixed infections with competing viruses. *N. clevelandii* plants, 8 or 10, were inoculated by hand rubbing with mixtures containing DNAs of pICPPV-VPgSwCM-R DNA and of one version of this chimera modified with the mutation F163L (**A**), P114S plus F163L (**B**) or P114S (**C**). Non-mutated/mutated chimera mixtures at 1.5:1 ratio, were employed for the three competitions. An additional 1:1 ratio mixture was used for the PPV-VPgSwCM-R vs P114S competition. Viral progenies were analyzed in pools of two plants by reverse transcription-polymerase chain reaction (RT-PCR) amplification and sequencing of a DNA fragment covering the VPg coding sequence. Images show the chromatograms of VPg codons 163 (position 2017–2019 in the viral genome) and/or 114 (position 1968–1970 in the viral genome). Viruses identified are indicated beneath the chromatograms; smaller letters indicate lower accumulation. A similar result was obtained in a replicate assay.

Next, competition assays were carried out using mixtures containing DNAs from the pICPPV-VPgSwCM-R plasmid and each of the mutants derived from it (P114S, F163L and P114S-F163L), at ratios in which the non-mutated chimera was over-represented (ratio 1.5:1) (Supplementary Figure S2C). *N. clevelandii* plants were inoculated by hand-rubbing and systemic infection was confirmed by visual inspection of symptoms and an anti-CP immunoblot assay (data not shown). Four pools, one per type of inoculum, were prepared by joining systemically infected tissue from two plants, collected at 21 dpi. A cDNA fragment covering the complete VPg sequence was amplified by IC-RT-PCR and sequenced (Figure 3). The results showed that, despite its lower representation in the inoculum, the mutant F163L was able to completely impose to the non-mutated chimera in all analyzed plants (Figure 3A). A similar result was observed after confrontation between the double mutant and the non-mutated chimera. In this case, the double mutant entirely prevailed in the progeny from three of pools, and it was in progress to do it in the fourth sample (Figure 3B). The result derived from competition between P114S and the non-mutated chimera was more even. The virus with the original VPg sequence was imposed in one of the four analyzed pools, while the P114S mutant entirely prevailed in two samples and coexisted with advantage in a third case (Figure 3C). The result of this competition, in which the mutant was underrepresented in the inoculum, ruled out that the P114S mutation reduces the virus fitness in *N. clevelandii*, and suggested that, in fact, it increases fitness. To further support this assumption, a second competition using comparable amounts of both viruses (ratio 1:1) was done (Supplementary Figure S2C), and the viral progeny was analyzed in ten plants, distributed in five pools. The results were in line with previous findings. This time, the mutant P114S was completely dominant in one pool and the majority in the remaining four, largely prevailing the non-mutated virus in two of them (Figure 3C).

Overall, these results indicate that specific mutations of VPg, in principle needed for adaptation in *A. thaliana*, do not provoke an adverse trade-off in *N. clevelandii*. On the contrary, these VPg changes appear to boost PPV fitness in two unrelated hosts.

The competition experiments in *A. thaliana* reported above did not reveal significant synergistic or additive effects of the P114S and F163L mutations in that host. Additional competition tests were conducted to assess possible accumulative effects of these mutations in *N. clevelandii.* Following an identical procedure to the aforementioned, viral progenies of eight *N. clevelandii* plants, distributed in four pools, were analyzed (Supplementary Figure S4). In the competition between F163L and P114S-F163L, the double mutant, underrepresented in the inoculum, did not outcompete the single mutant in any of the samples analyzed. The P114S-F163L mutant was able to reverse its underrepresentation when competing with the P114S single mutant, reflecting that a greater fitness gain could be associated to F163L mutation. However, such a difference would be very subtle because the P114S mutant was completely or almost completely imposed in two samples (Supplementary Figure S4). Thus, in *N. clevelandii*, the joint presence of the two mutations in the P114S-F163L mutant does not appear to entail a relevant fitness increase with respect to any of individual F163L or P114S mutations.

### 3.3. Changes in VPg Protein Resulting from Adaptation to Arabidopsis thaliana Prompt PPV-VPgSwCM-R infection in Chenopodium foetidum

Most PPV isolates, including PPV-R (D strain) induce necrotic local lesions in *C. foetidum*, compatible with a hypersensitive-like response. In contrast, isolates from strain C, in particular the isolate PPV-SwCMp, cannot infect this host, thus emulating what happens in *A. thaliana* [44]. Similarly, the defect of chimera PPV-VPgSwCM-R (VPg from PPV-SwCMp in the backbone of PPV-R, Supplementary Figure S1) in *A. thaliana* is extensible to *C. foetidum* [44]. Having demonstrated that mutations P114S or F163L at the VPg protein of PPV-SwCMp enable to rescue infectivity of PPV-VPgSwCM-R in *A. thaliana*, we decided to check the effect of these mutations on the infectivity of this chimera in *C. foetidum*.

First, we tested whether the adaptation of PPV-VPgSwCM-R to *A. thaliana* facilitated infection of *C. foetidum*. For this purpose, *C. foetidum* leaves were inoculated by hand-rubbing with leaf extracts from *N. clevelandii* plants infected with viral progenies of the PPV-VPgSwCM-R chimera adapted to *A. thaliana* upon introducing VPg mutations P114S or F163L. As controls of infection in this host, *C. foetidum* leaves were also inoculated with extracts of *N. clevelandii* plants infected with PPV-R, PPV-SwCMp or the non-mutated chimera PPV-VPgSwCM-R (Supplementary Figure S5A). To warrant delivery of same virus amounts, inoculum concentrations were adjusted by dilution with extracts from healthy *N. clevelandii* (Supplementary Figure S5B). PPV-R caused a large number of lesions (more than 60 per leaf) that rapidly necrotized, causing death and dropping of the leaves sometimes before 9 dpi. As expected, no lesions were observed in leaves inoculated with PPV-SwCMp. *C. foetidum* leaves inoculated with the extracts containing the evolved PPV-VPgSwCM-R populations, displayed abundant lesions, although in lesser amount than in those inoculated with PPV-R: at 9 dpi, approximately 6.5 and 17 per leaf for the chimeras with the P114S and F163L mutations, respectively. Moreover, although some of the lesions caused by the evolved chimeras necrotized, in general they were less severe than those caused by PPV-R, remaining alive the inoculated leaves even after 15 dpi (Supplementary Figure S5C and Supplementary Table S2). To assess whether the virus further evolved in *C. foetidum*, viral progenies of individual lesions were amplified in *N. clevelandii*, and their VPg sequences were determined after IC-RT-PCR amplification. No changes beyond original modifications introduced during adaptation in *A. thaliana* were detected in any of 4 *N. clevelandii* plants analyzed. A few spots suspected of being viral lesions were observed in leaves inoculated with the non-mutated PPV-VPgSwCM-R, however, we were not able to infect *N. clevelandii* with them, suggesting that either they had not been correctly identified, or their viral load was very low.

**Figure 4:**
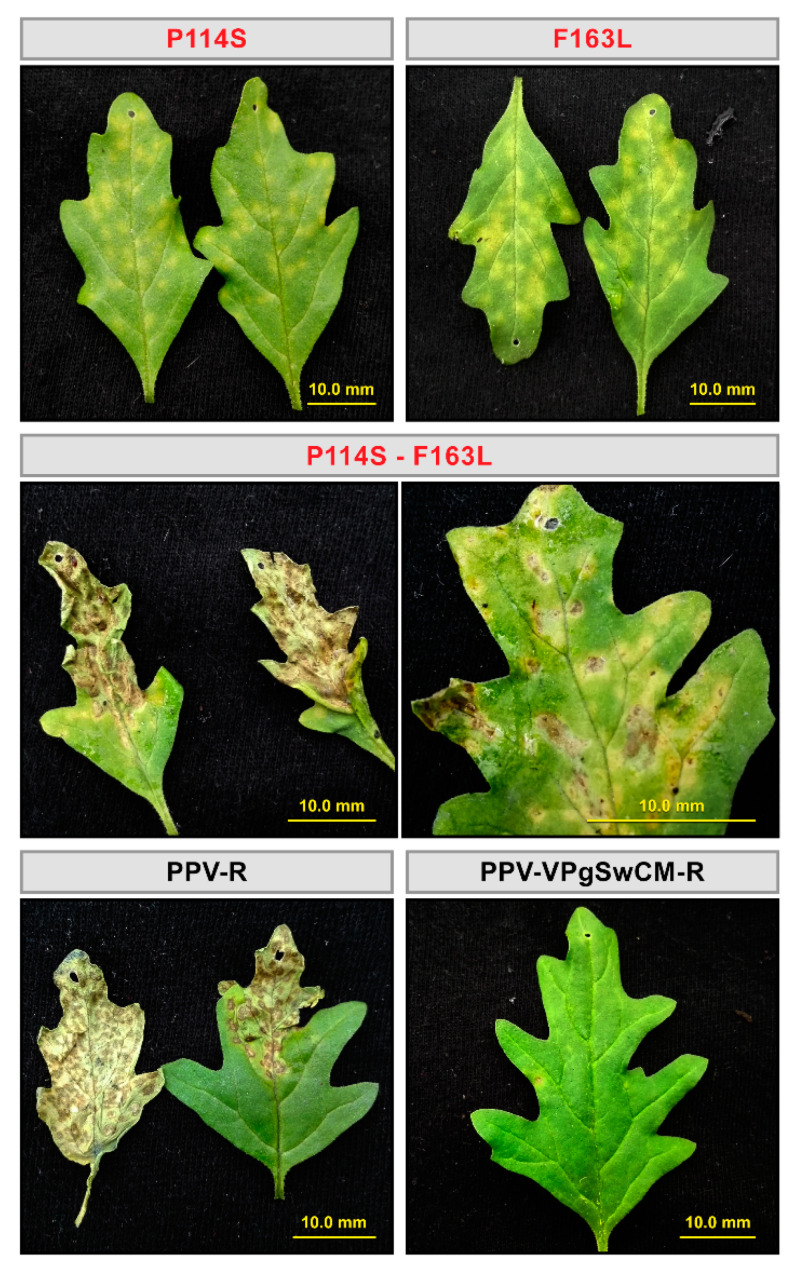
Effect of VPg mutations on *Chenopodium foetidum* infection by *Plum pox virus* (PPV). *C. foetidum* leaves were inoculated by hand rubbing with leaf extracts of *Nicotiana clevelandii* plants, previously infected with two independent clones of pICPPV-VPgSwCM-R, variants of this chimeric clone mutated as indicated, or pICPPV-NK-lGFP (PPV-R isolate). Eight plants (three leaves per plant) per construct (four per clone) were inoculated. Representative images taken at 12 dpi under visible light are shown. Bar, 10.0 mm.

To verify that the apparent adaptation of the PPV-VPgSwCM-R to *C. foetidum* was in fact due to the VPg mutations P114S and F163L, the exclusive contribution of these substitutions was assessed making use of corresponding mutated cDNA clones (Supplementary Figure S1). pICPPV-VPgSwCM-R and its mutated forms (P114S, F163L and P114S-F163L), as well as the PPV-R cDNA clone pICPPV-NK-lGFP were manually inoculated in *N. clevelandii* plants by hand-rubbing. Leaf extracts from these infected plants were in turn inoculated into *C. foetidum* leaves by hand-rubbing, after adjusting its concentration with extract of healthy *N. clevelandii* to warrant delivery of same virus amounts (Supplementary Figure S6). Both mutants, P114S and, in a greater extent, F163L caused abundant local lesions (Table 1 and Figure 4). As in the above experiment using the non-cloned mutant viruses, lesions caused by both mutants were similar and milder than those triggered by PPV-R. Interestingly, simultaneous presence of both mutations fostered a qualitative change in viral symptoms, as reflected in the PPV-R-like lesions induced by the P114S-F163L mutant (Table 1 and Figure 4). Some potential lesions were also detected in a few leaves inoculated with the non-mutated pICPPV-VPgSwCM-R chimera, mainly at 17 dpi, when all leaves inoculated with the rest of viruses were dead (Table 1). Viral cDNA from some suspicious lesions could be amplified by IC-RT-PCR, and subsequent sequencing showed that the wild type VPg sequence had been maintained in five analyzed viral progenies. These results confirmed that, although the PPV-VPgSwCM-R chimera was not completely unable to infect *C. foetidum*, its ability to infect this host is greatly enhanced by VPg mutations selected during adaptation to *A. thaliana.*

### 3.4. Sequence Heterogeneities Between Nicotiana clevelandii, Arabidopsis thaliana and Chenopodium foetidum eIF(iso)4Es map to the eIF4E/VPg Interface

The dysfunction of SwCMp VPg-containing PPV chimera in *A. thaliana* was suggested to be caused by a defect in VPg/eIF(iso)4E interaction, because mutations promoting infection in this host resembled those detected in potyviruses escaping eIF4E/(iso)4E- based resistance [44]. Since the mutations selected in *A. thaliana* also boosted infection in *C. foetidum*, we speculated that common characteristics of eIF(iso)4E factors of these two species, not shared by *N. clevelandii* eIF(iso)4E, prevented interaction with SwCMp VPg, and, thus, the infection by SwCMp VPg-containing PPV.

Recently, it has been reported the first high-resolution structure of the VPg from a potyvirus, PVY [51]. This study not only revealed interesting structural data concerning the VPg folding, but also identified residues implicated in its interaction with an eIF4E factor. In light of this information, we decided to extrapolate available interaction data of PVY VPg and eIF4E to the proteins subject of interest for our work, by examining heterogeneities in the primary sequence of PPV VPg variants and those of eIF(iso)4Es from the three herbaceous hosts here studied.

**Figure 5:**
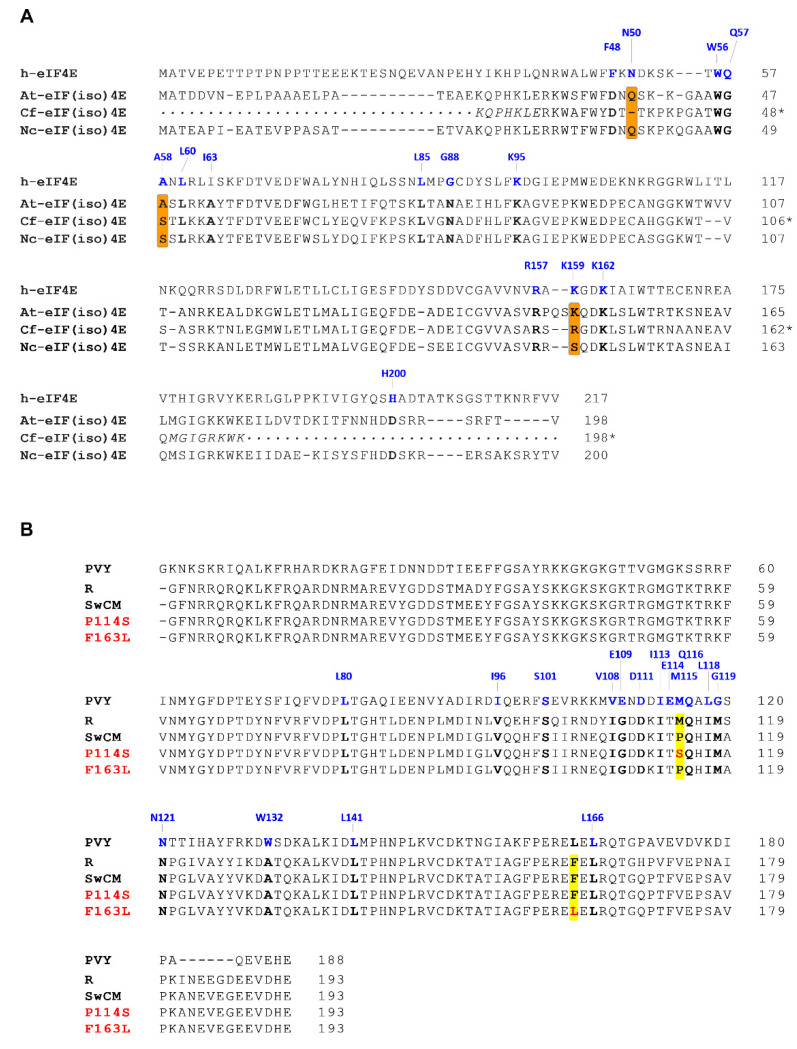
(**A**) Alignment of the translation initiation factors 4E from *Homo sapiens* (h-eIF4E) (P06730.2) along with eIF(iso)4Es from *Arabidopsis thaliana* [At-eIF(iso)4E] (O04663.2), *Chenopodium foetidum* [Cf-eIF(iso)4E] (partial sequence specifically obtained for this study) and *Nicotiana clevelandii* [Nc-eIF(iso)4E] (KC625579.1). Residues of eIF(iso)4E plant factors aligning with those interacting residues of h-eIF4E appear in bold and, in case of no conservation among the three plant species, are orange highlighted. Amino acids encoded by primers used for PCR amplification of the Cf-eIF(iso)4E sequence are italicized. Asterisks in the numbering of the partial Cf-eIF(iso)4E sequence indicate that for the count, missing amino acids were replaced by the equivalent ones from the full-length sequence of the *Chenopodium quinoa* eIF(iso)4E protein (LOC110697254 and LOC110692931). (**B**) Alignment of VPg sequences from *Potato virus Y* (PVY) (QED90173.1) and *Plum pox virus* (PPV), R isolate (EF569215) and SwCMp isolate (SHARCO database, http://w3.pierroton.inra.fr:8060, accessed on 1 April 2021). Two mutations independently arisen at SwCMp VPg, as consequence of the adaptation in *A. thaliana*, are shown in red over a yellow-shaded box. In both panels, specific amino acids perturbed as a result of interaction between h-eIF4E and PVY VPg, according to Coutinho de Oliveira et al. [51], are highlighted in blue. Residues of PPV VPg aligning with the h-eIF4E -interacting PVY VPg residues appear in bold. Protein sequences were aligned using Clustal Omega program (European Bioinformatics Institute), then adjusted by minor manual corrections.

The eIF(iso)4E sequences of *N. clevelandii* [Nc-eIF(iso)4E] and *A. thaliana* [At-eIF(iso)4E] were retrieved from NCBI protein database, but that of *C. foetidum* [Cf-eIF(iso)4E] was not available in public databases and had to be specifically obtained for this analysis. Total RNA of *C. foetidum* was retrotranscribed to prepare cDNA, from which to amplify a fragment encoding a segment of the Cf-eIF(iso)4E, by using a pair of degenerated primers designed based on the eIF(iso)4E sequence of *C. quinoa*. The amplicon covered the regions identified as relevant for the interaction with VPg by Coutinho de Oliveira et al. [51]. Alignment of the three eIF(iso)4E sequences (Figure 5A) revealed that Cf-eIF(iso)4E was not more similar to At-eIF(iso)4E than to Nc-eIF(iso)4E; in fact, the level of identity with the second protein was slightly higher than with the first one (77.3% vs 73.8%).

Following, we focussed more closely on regions that are involved in eIF4E/VPg interactions according to data obtained from a complex formed among the human eIF4E (h-eIF4E) and PVY VPg, reported by Coutinho de Oliveira et al. [51]. The main interface between these two proteins embraces the cap binding pocket of eIF4E, and includes several residues shown to be perturbed by VPg binding (F48, N50, W56, Q57, A58, L60, G88, R157, K159 and K162), and a VPg loop containing residues that are affected by interaction with eIF4E (V108, E109, D111, I113, E114, M115, Q116, L118, G119 and N121) (Figure 5 and Supplementary Figure S7). When we scrutinize the alignment of the eIF(iso)4Es of *A. thaliana*, *C. foetidum* and *N. clevelandii* along with h-eIF4E, we observed sequence heterogeneities between the three plant eIF(iso)4Es at three positions that align with some of the h-eIF4E amino acids directly involved in interaction with PVY VPg: N50, A58 and K159. However, eIF(iso)4Es of *A. thaliana* and *C. foetidum* only clustered together at the position equivalent to that occupied by K159 in h-eIF4E (see K and R in At-eIF(iso)4E and Cf-eIF(iso)4E, respectively) leaving apart the *N. clevelandii* protein [see S in Nc-eIF(iso)4E] (Figure 5A). Interestingly, one of the VPg mutations facilitating PPV-VPgSwCM-R adaptation to *A. thaliana* and *C. foetidum*, P114S, falls into the VPg loop that interacts with the eIF4E cap-binding domain. This residue is equivalent to PVY M115 (Figure 5B), proposed to form part of a hydrophobic pocket that buries W56 [51], a residue of h-eIF4E close to A58, in turn aligned with a polymorphic position of plant eIF(iso)4Es (Figure 5A). This observation suggests that species-specific features governing eIF4E/(iso)4E-VPg interactions at this region are important for susceptibility to different potyvirus variants. However, the lack of a positive correlation between susceptibility to non-mutated PPV-VPgSwCM-R and the clustering of such a polymorphic residue (A in *A. thaliana* and S in both *N. clevelandii* and *C. foetidum* (Figure 5A), precludes drawing straightforward conclusions.

The other VPg mutation associated to PPV-VPgSwCM-R adaptation to *A. thaliana* and *C. foetidum*, F163L, appears to be far from the protein-protein interface defined for PVY VPg and h-eIF4E (Supplementary Figure S7). However, it is very close to PVY L166 (Figure 5B), a neighboring residue to a flexible loop between two ß strands, that has been identified to be perturbed by eIF4E binding [51]. Thus, it is tempting to speculate that this target, outside the cap-binding site/VPg interface, is involved in species-specific interactions still to be characterized.

## 4. Discussion

Viral cross-species jump takes place once a virus develops the ability to infect, replicate and disseminate among individuals of a new host species [3,52]. This phenomenon has been more frequently described for RNA viruses, mainly among *Rhabdoviridae* and *Picornaviridae* family members, and it has been associated with its huge adaptive plasticity [53,54]. From the human health perspective, some of the more remarkable examples of zoonotic RNA viruses able to break interspecies barrier by the assistance of an intermediate host are *influenza A virus* (IAV)*, human immunodeficiency virus* (HIV) and several respiratory coronaviruses [55,56,57]. Spillover events are also quite often in plant viruses, linked to multiple factors, including ecological conditions, genetic plasticity of virus components, and host factor requirements [8,58]. Genome nature appears to be one of the elements that determine the capacity of the virus to successfully infect a host variety; and among plant viruses, single-stranded RNA genome viruses are those with largest host-range breadth [9].

In this study, we have delved into the contribution of two adaptive changes that affect the VPg protein sequence by promoting infectivity gain of a potyvirus in two non-permissive hosts. Potyviral VPg has a high content of intrinsically disordered regions, a characteristic associated with larger mutational robustness which favourably impacts on viral adaptive plasticity [21,26]. Here, we took advantage of the PPV chimeric clone pICPPV-VPgSwCM-R that bears the VPg sequence of PPV-SwCMp, an isolate unable to infect *A. thaliana,* in the backbone of the infectious PPV-R isolate [44]. We specifically studied infections in different hosts triggered by mutations at this chimeric clone that were engineered on the basis of VPg changes known to emerge during adaptation of PPV-VPgSwCM-R to *A. thaliana* [44]. Our results confirm that any of these two mutations, P114S or F163L, is sufficient to prompt the break of resistance to PPV-VPgSwCM-R in *A. thaliana* (Figure 1).

More important, the mutations selected to adapt the chimera to *A. thaliana* were also able to boost the infection in a second resistant species, *C. foetidum.* Although PPV-VPgSwCM-R is very poorly infectious in both *A. thaliana* and *C. foetidum*, it is still able to carry out a basal replication in both restrictive hosts as evidenced by the emergence of adaptive mutations in *A. thaliana* and late-onset sporadic lesions in *C. foetidum.* VPg mutations selected in *A. thaliana* promote viral infection in both hosts, but, while in *A. thaliana* the mutant viruses reach amplification levels similar to those of well-fitted PPV-R isolate, the infection they cause in *C. foetidum* is considerably milder than that induced by PPV-R. This indicates that mutations facilitating functional interactions of VPg with *A. thaliana* host factors, also improve matching with the homologous factors of *C. foetidum*, but to a lesser extent (Figure 1, Figure 4, Table 1, Supplementary Figure S5, Supplementary Table S2).

Although the double mutation P114S-F163L was not detected in the natural adaptation of PPV-VPgSwCM-R to *A. thaliana*, we also engineered it in pICPPV-VPgSwCM-R. No additive or synergistic effects were observed in *A. thaliana*, where the double mutation seemed to confer a little better fitness than the single mutation P114S, but did not improve the F163L performance. In contrast to the effect of the single mutations, concurrence of the two mutations had a differential impact over the typology of lesions caused by the viral chimera in *C. foetidum*, making them similar to those produced by PPV-R. This result further supports the assumption that the effect of P114S and F163L mutations on the coupling between PPV VPg and host-specific plant cofactors is different in *A. thaliana and C. foetidum* (Figure 4, Table 1).

The nature of the mutations favouring the adaptation of PPV-VPgSwCM-R to *A. thaliana* led Calvo et al. [44] to conclude that resistance to PPV-SwCMp in *A. thaliana* and *C. foetidum* is due to incompatible interactions between PPV VPg and plant eIF(iso)4Es. The establishment of a productive interaction between VPg and eIF4E factors or its isoforms is critical for potyviral infection, as demonstrated by many studies connecting specific mutations in VPg with resistance breakdown events, and numerous examples of eIF4E-mediated plant resistance against potyviruses [27,28,30,59]. In this sense, the recently solved potyviral VPg structure and the characterization of a VPg-eIF4E complex have shed light on this issue [51]. By using the HADDOCK eIF4E-VPg model generated in that work, we got positional information about the two mutation targets linked to SwCMp VPg adaptation (Supplementary Figure S7). We observed that one of these targets (P114 in PPV-SwCMp, S114 in the adapted mutant and M114 in PPV-R) is equivalent to the residue M115 of PVY VPg, which maps to the interface connecting both interacting molecules and whose involvement in such interaction had been experimentally validated by Coutinho de Oliveira et al. [51]. Indeed, PVY M115 is proposed to form part of a hydrophobic pocket in which a specific residue of the cap binding domain of h-eIF4E (W56) is buried. This residue is spatially close to another involved in the interaction, A58 of h-eIF4E, equivalent to an amino acid varying among the three analyzed plant eIF(iso)4E factors, *A. thaliana* (A48), *C. foetidum* (S49) and *N. clevelandii* (S50) (Figure 5A,B). Interestingly, also positions equivalent to 159 of h-eIF4E, included in the positive patch R157-K159-K162 that interacts with VPg negative amino acids, also at the interface [51], exhibit variability among the three herbaceous eIF(iso)4E sequences (Figure 5A). The lack of conservation suggests these regions would confer host-specific interaction performances on the protein. Thus, it is plausible that P114S mutation aims to achieve a more optimal fit of VPg, meeting particular requirements for the formation of a complex containing *A. thaliana* eIF(iso)4E. The apparent incongruence found when analyzing sequences of the eIF(iso)4E factors from permissive *N. clevelandii* (S50 and S147) and non-permissive *A. thaliana* (A48 and K149) and *C. foetidum* (S49 and R146) species (Figure 5A) could be explained by the fact that it is the functional capacity, rather than the primary sequence of the eIF4E/(iso)4E plant factors, what decides whether a successful infection takes place. Something similar was reported by Estevan et al. [60], who observed that, although *Tobacco etch virus* (TEV) uses *A. thaliana* eIF(iso)4E as cofactor, a convenient *trans*-complementation occurs by supplying *Capsicum annuum* eIF4E instead of *C. annuum* eIF(iso)4E.

The second mutation allowing adaptation to *A. thaliana*, F163L, affects a residue that does not match any of the PVY VPg amino acids located in the interface with h-eIF4E, as determined by Coutinho de Oliveira et al. [51]. However, experimental data obtained from that work showed that the PVY VPg amino acid L166, equivalent to a close neighbor of the mutated target of PPV VPg, was perturbed by h-eIF4E binding (Supplementary Figure S7).

These observations suggest that PPV-SwCMp VPg can adapt to At-eIF(iso)4E by two, probably independent, mechanisms. First, through P114S mutation, by improving the interaction of a VPg eIF4E-binding domain with the cap-binding domain of eIF(iso)4E. The second substitution, mediated by F163L, is less obvious. Although we cannot discard the occurrence of long-distance allosteric interactions between the residue 163 and VPg/eIF4(iso)4E cap binding domain interface, it seems more likely that this amino acid might participate in interactions not identified by Coutinho de Oliveira et al. [51] due to intrinsic limitations of their model. In this respect, it is important to remark that in the work of these authors, the VPg structure was obtained from a bacterial-expressed protein lacking the first 37 amino acids. Besides, the VPg-eIF4E complex was generated using the human factor eIF4E, and in absence of other suggested partners, both from the plant [eIF(iso)4G] and from the virus (P1, HCPro, CI) (see Section 1). It is clear that, in spite of the high value of the model reported by Coutinho de Oliveira et al. [51], more sophisticated studies are required to ascertain the structural details determining the compatibility spectrum between potyviral VPgs and host eIF4E/(iso)4E cofactors.

An important conclusion derived from our work points out to the necessary intervention of an intermediate host to prompt the adaptation to a second non-related host. As mentioned before, sporadic and mild infections by the non-mutated chimera PPV-VPgSwCM-R were detected in *C. foetidum*; but it is only through mutations affecting specific amino acids of SwCMp VPg after adaptation in *A. thaliana* that a robust infection in *C. foetidum* is triggered (Figure 4, Table 1, Supplementary Figure S5, Supplementary Table S2). The dynamizing role of intermediate bridge species in virus host range expansion has been described in animal systems, being especially relevant in cases of global pandemics involving animal-to-humans virus jumping [61,62,63]. In plants, adaptation of a virus to a particular host can have expanding ecological consequences once enabling adaptation to related plants. This has been described for interactions of PVY and plants of the *Solanaceae* family, in which PVY mutations that break the resistance generated by a particular eIF4E allele concurrently confer adaptation to additional plant genotypes with different eIF4E alleles [64]. Our results indicate that a bridge host can also help to break interfamily barriers, which are assumed to frequently restrict the viral host range expansion [9]. The reason why PPV-VPgSwCM-R cannot adapt by itself to *C. foetidum* and needs pre-adaptation in *A. thaliana* is probably because local lesions cause bottlenecks preventing fitness gain via natural selection [65,66]. However other obstacles, mainly genetic, but also ecological, can make bridge species especially necessary for some host jumpings.

Jumping to new hosts usually brings an adaptive cost in the initial host [12]. However, adaptation of PPV-VPgSwCM-R to *A. thaliana* does not seem to imply an adverse trade-off in the previously-adapted host *N. clevelandii* (Figure 3). There are previous reports showing fitness losses driven by PPV mutations that are associated to woody-to-herbaceous host jumpings [46,67,68]. And there are also examples in which the adaptation of PPV isolates to herbaceous plants did not seem to affect its ability to infect the *Prunus* species from which they came [68,69]. However, in these studies a limited trade-off linked to the jump cannot be rule out, as no competition experiments or fitness quantifications have been conducted in them. In our case, the adaptive mutations at SwCMp VPg, selected in *A. thaliana*, not only do not impose a trade-off in *N. clevelandii*, but even confer some better fitness in this host. This observation suggests that adaptation of PPV-R and PPV-SwCMp to *N. clevelandii* is not optimal; probably because these isolates have been replicating in *N. clevelandii* for a long time in human terms, but too short on an evolutionary scale, so they have not been able to fix mutations that provide small fitness gains.

Overall, this study aims to highlight the importance of bridge hosts, exposing the possibility that, as in our case, certain adaptive changes not only contribute to the expansion of the host range as a consequence of the initial jump, but additionally they allow distant species, directly inaccessible, to become regular hosts. In short, these “encounters” with one or more “appropriate intermediaries” could act as shortcuts, radically facilitating the way in which a virus maximizes its host range.

## Figures and Tables

**Table 1:** Effect of VPg mutations on the infection of the PPV-VPgSwCM-R chimera in *Chenopodium foetidum.*

Inoculum	Total Inoculated Leaves	Number of Lesions ^a^	
		10 dpi	17 dpi
**PPV-VPgSwCM-R**	24	1 ^chl^ (1) 4 ^?^ (3)	4 ^chl^ (3) 1 ^nec^ (1) 10 ^?^ (6)
**P114S**	24	330 ^chl^ (24)	DL (24)
**F163L**	24	400 ^chl^ (24)	DL (24)
**P114S-F163L**	24	255 ^chl/nec^ (15) 145 ^nec^ (8) DL (1)	DL (24)
**PPV-R**	12	170 ^nec^ (10) DL (2)	DL (24)

^a^ The numbers of lesions are approximate because many of them merged at indicated days post inoculation (dpi). The number of leaves is indicated in parenthesis, specifying the different type of lesions: ^chl^, chlorotic; ^nec^, necrotic; ^?^, atypical. DL indicates death leaves.

## Data Availability

Figure 1, Figure 2, Figure 3, Figure 4 and Figure 5 and Table 1 appear in colour online. Additional supporting information may be found in the online version of this article. 7 Supplementary Figures and 2 Supplementary Tables are published online.

## Supplementary Materials

The following are available online at https://www.mdpi.com/article/10.3390/microorganisms9040805/s1, Figure S1: Sequence of VPg in the polyproteins of the chimeric construct pICPPV-VPgSwCM-R [44] and its mutated versions. Polyprotein regions derived from *Plum pox virus* (PPV) isolates R and SwCMp are shown in grey and orange, respectively. Underlined amino acids in the VPg protein of SwCMp were mutated to those depicted in red. Figure S2: Schematic representation of the experimental approach followed to assess the effect of VPg mutations on *Plum pox virus* (PPV) infection in different hosts. **(A)** Full-length PPV cDNA clones were mechanically inoculated into *Arabidopsis thaliana* plants by biolistic (represented by a Gene Gun device), or into *Nicotiana clevelandii* and *Chenopodium foetidum* plants by hand-rubbing (represented by a Carborundum bottle). **(B,C)** Competitions assays between single and double mutants in *A. thaliana* and *N. clevelandii* **(B)** or between non-mutated chimera and VPg mutants in *N. clevelandii* **(C)** were carried out using eight plants (P1–P8) and mixtures of DNAs at the specified ratios. Amounts of DNAs used as inocula were adjusted to deliver each virus at specified doses, as the enzymatic pattern rendered after *EcoR*I digestion of each construct shows. Fragments yielded by the *Hind*III-digested ø29 fago DNA used as molecular-weight size marker (M) are also indicated. Figure S3: Effect of VPg mutations on *Plum pox virus* (PPV) infection of *Nicotiana clevelandii*. *N. clevelandii* plants were inoculated by hand-rubbing with DNAs of the chimeric clone pICPPV-VPgSwCM-R, the indicated chimera-derived mutants (two independent clones, 1 and 2), or the PPV-R clone pICPPV-NK-lGFP. Extracts from upper non-inoculated leaves collected at 15 days after inoculation were subjected to CP-specific immunoblot analysis. Two individual plants (P1 and P2) inoculated with the specified viruses, were analysed. Blots stained with *Ponceau* red showing the large subunit of the ribulose-1,5-bisphosphate carboxylase/oxygenase (RuBisCO) are included as loading controls. Figure S4: Sequence analysis of viral progeny from *Nicotiana clevelandii* exposed to mixed infections with competing viruses. Eight *N. clevelandii* plants were inoculated by hand-rubbing with DNA mixtures containing the indicated pICPPV-VPgSwCM-R-derived mutant clones. In the two competitions, the single mutant was overrepresented in the inoculum with respect to the double mutant (ratio 1.5:1). Viral progenies were analysed in pools of two plants by reverse transcription-polymerase chain reaction (RT-PCR) and sequencing of a cDNA fragment covering the VPg coding sequence. Images show the chromatograms of VPg codons 114 (position 1968–1970 in the viral genome) **(A)** or 163 (position 2017–2019 in the viral genome) **(B)**. Viruses identified are indicated beneath the chromatograms; smaller letters indicate lower accumulation. Figure S5: Effect of VPg mutations on *Nicotiana clevelandii* infection by *Plum pox virus* (PPV). **(A)** Schematic representation of the experimental approach. **(B)** Prior to *C. foetidum* inoculation, extracts from upper non-inoculated leaves of *N. clevelandii* plants infected with the different viruses, collected at 10 days after inoculation, were subjected to coat protein (CP)-specific immunoblot analysis to estimate virus accumulation, thus allowing to adjust the inoculum doses. Increasing dilutions of the extracts from plants infected with the mutant viruses are indicated. The lower signal observed for the PPV-SwCMp sample is a consequence of the significantly poorer recognition of PPV-SwCMp CP compared to PPV-R CP by the choice antibody. Blots stained with *Ponceau* red showing the large subunit of the ribulose-1,5-bisphosphate carboxylase-oxygenase (RuBisCO) are included as loading controls in both cases. **(C)** Images of *C. foetidum* leaves taken under visible light at 15 days post infection, after being inoculated with extracts of infected *N. clevelandii* plants. Bar, 5 mm. Figure S6: Assessment of viral titers in *Nicotiana clevelandii* extracts employed to inoculate *Chenopodium foetidum* plants. *N. clevelandii* plants were inoculated by hand-rubbing with DNA of the chimeric clone pICPPV-VPgSwCM-R, the indicated chimera-derived mutants (two independent clones, 1 and 2), or the PPV-R clone pICPPV-NK-lGFP. Extracts were prepared from upper non-inoculated leaves collected at 7 days post inoculation and their virus titers were adjusted with extracts from healthy leaves on the basis of a previous quantitative anti-CP immunoblot assay. The adjusted extracts were inoculated by hand-rubbing in *C. foetidum* leaves. Equalization of viral titers in the inocula was verified in the CP-specific immunoblot shown in the figure. Blots stained with *Ponceau* red showing the large subunit of the ribulose-1,5-bisphosphate carboxylase-oxygenase (RuBisCO) are included as loading controls. Figure S7: HADDOCK-derived structure of the human eIF4E (h-eIF4E) (blue molecule) in complex with the VPg of *Potato virus Y* (PVY) (PVY VPg) (red molecule), obtained by Countinho de Oliveira et al. [51], in which the following residues are highlighted: (i) PVY VPg residues perturbed by h-eIF4E binding (in salmon); (ii) residues in PVY VPg equivalent to those of *Plum pox virus* VPg mediating adaptation of the chimeric virus PPV-VPgSwCM-R to *Arabidopsis thaliana* and *Chenopodium foetidum*, P114 [that match one amino acid specified in (i)] and F163 (both in red); (iii) h-eIF4E residues perturbed by PVY VPg binding, whose equivalents are conserved among *A. thaliana*, *Nicotiana clevelandii* and *C. foetidum* (in cyan); and iv) residues in h-eIF4E perturbed by PVY VPg binding, whose equivalents in eIF(iso)4E from *A. thaliana*, *N. clevelandii* and *C. foetidum* are not fully conserved (in bright blue). Structural details were visualized using PyMOL. On the left, h-eIF4E:PVY VPg complex, pointing key residues addressed in this study. On the right, both molecules, separately disposed, indicating all residues reported to be perturbed after protein:protein interaction, according to Counthino de Oliveira et al. [51]. Table S1: Primer list. Table S2: Effect of specific mutations at the VPg of the *Plum pox virus*, isolate SwCMp (strain C), on the infection of *Chenopodium foetidum.*
