# Supplementary material for: Virus Host Jumping Can Be Boosted by Adaptation to a Bridge Plant Species
Source: Microorganisms. 2021 Apr 11;9(4):805. doi: 10.3390/microorganisms9040805 (PMC8070427; doi:10.3390/microorganisms9040805)
Supplement: Supplementary file 1 [file microorganisms-09-00805-s001.zip › Supplementary_Material_100421_.pdf]

SUPPLEMENTARY MATERIALS

Supplementary Figure S1

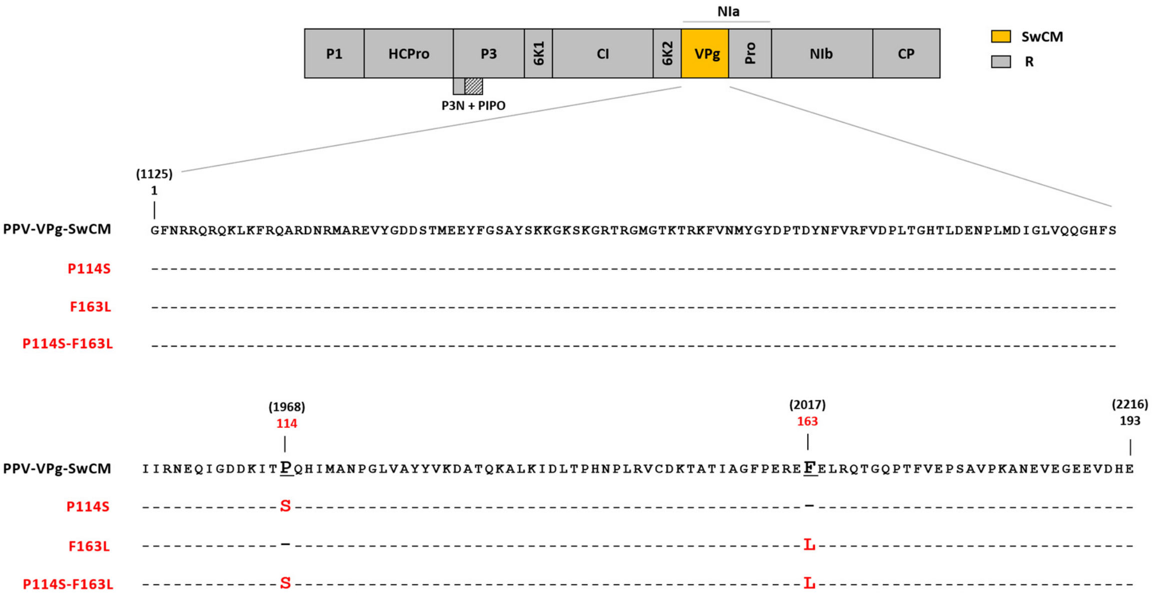

Supplementary Fig. S1: Sequence of VPg in the polyproteins of the chimeric construct pICPPV-VPgSwCM-R [44] and its mutated versions. Polyprotein regions derived from *Plum pox virus* (PPV) isolates R and SwCMp are shown in grey and orange, respectively. Underlined amino acids in the VPg protein of SwCMp were mutated to those depicted in red.

Supplementary Figure S2

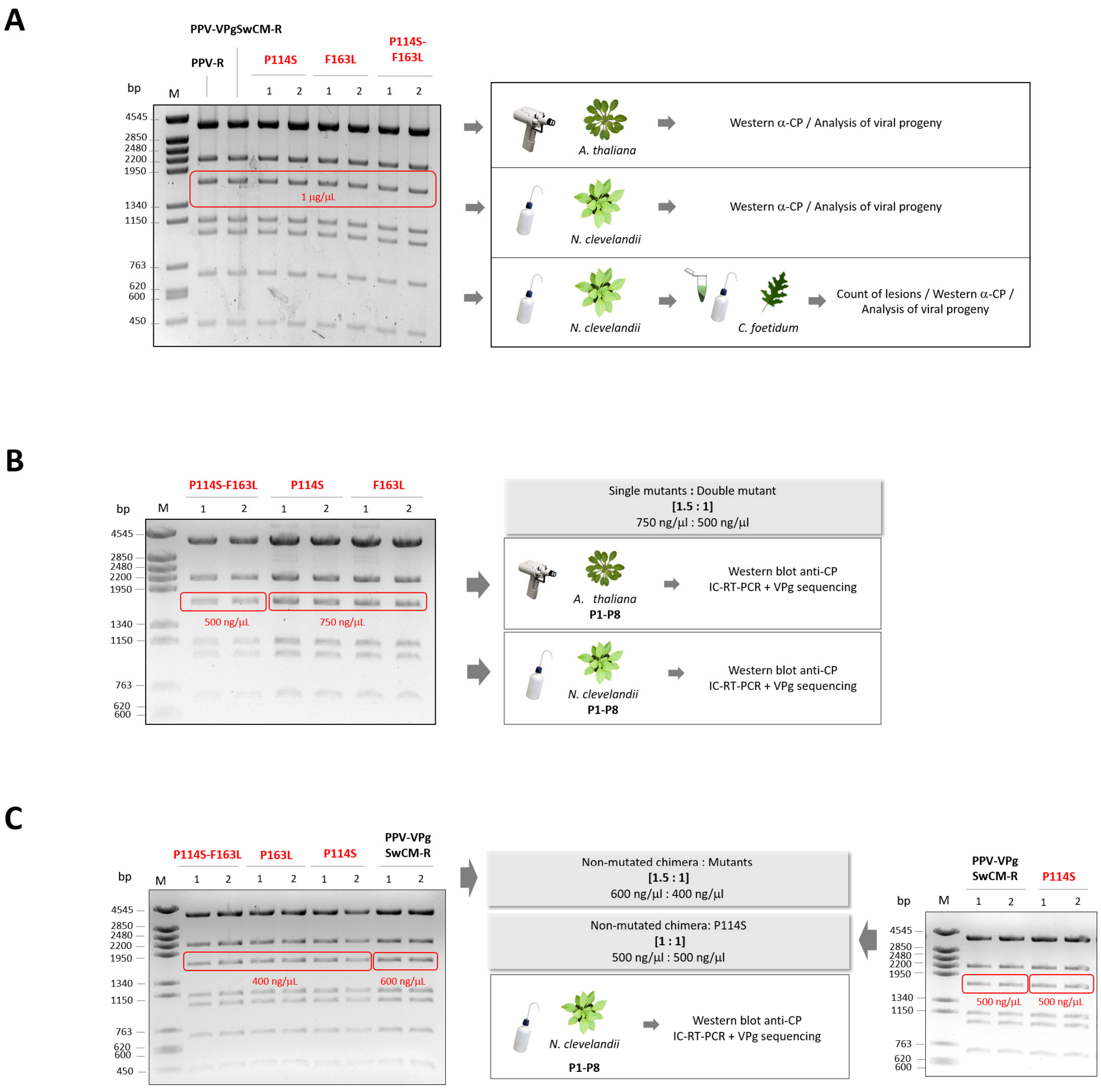

Supplementary Fig. S2. Schematic representation of the experimental approach followed to assess the effect of VPg mutations on *Plum pox virus* (PPV) infection in different hosts. **(A)** Full-length PPV cDNA clones were mechanically inoculated into *Arabidopsis thaliana* plants by biolistic (represented by a Gene Gun device), or into *Nicotiana clevelandii* and *Chenopodium foetidum* plants by hand-rubbing (represented by a Carborundum bottle). **(B-C)** Competitions assays between single and double mutants in *A. thaliana* and *N. clevelandii* **(B)** or between non-mutated chimera and VPg mutants in *N. clevelandii* **(C)** were carried out using eight plants (P1-P8) and mixtures of DNAs at the specified ratios. Amounts of DNAs used as inocula were

adjusted to deliver each virus at specified doses, as the enzymatic pattern rendered after *EcoR*I digestion of each construct shows. Fragments yielded by the *Hind*III-digested ϕ29 fago DNA used as molecular-weight size marker (M) are also indicated.

Supplementary Figure S3

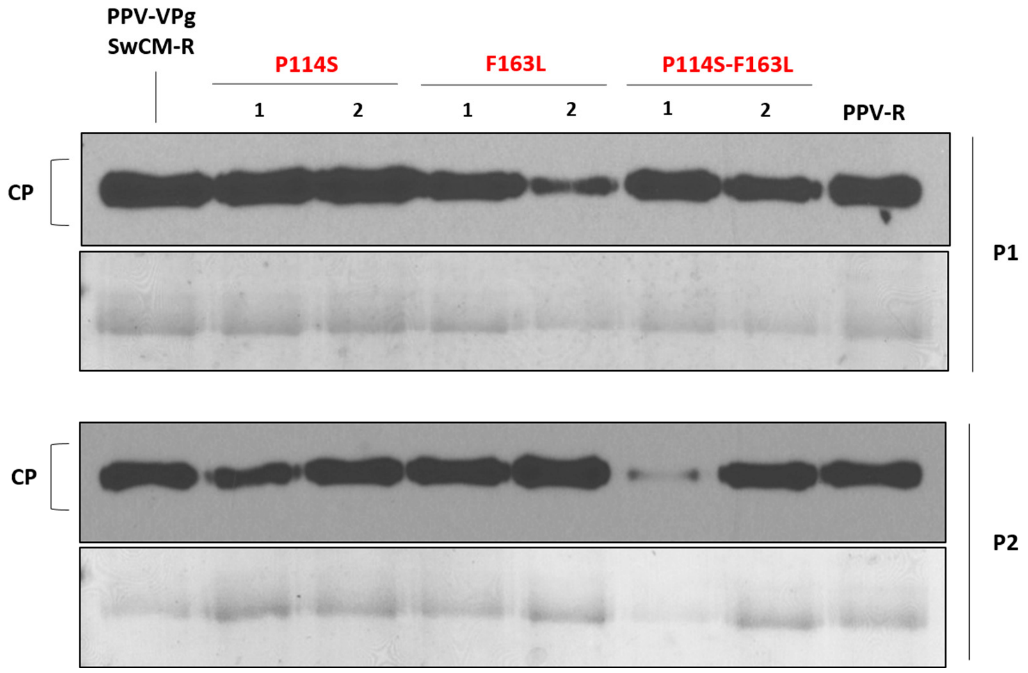

Supplementary Fig. S3: Effect of VPg mutations on *Plum pox virus* (PPV) infection of *Nicotiana clevelandii*. *N. clevelandii* plants were inoculated by hand-rubbing with DNAs of the chimeric clone pICPPV-VPgSwCM-R, the indicated chimera-derived mutants (two independent clones, 1 and 2), or the PPV-R clone pICPPV-NK-lGFP. Extracts from upper non-inoculated leaves collected at 15 days after inoculation were subjected to CP-specific immunoblot analysis. Two individual plants (P1 and P2) inoculated with the specified viruses, were analysed. Blots stained with *Ponceau* red showing the large subunit of the ribulose-1,5-bisphosphate carboxylase/oxygenase (RuBisCO) are included as loading controls.

Supplementary Figure S4

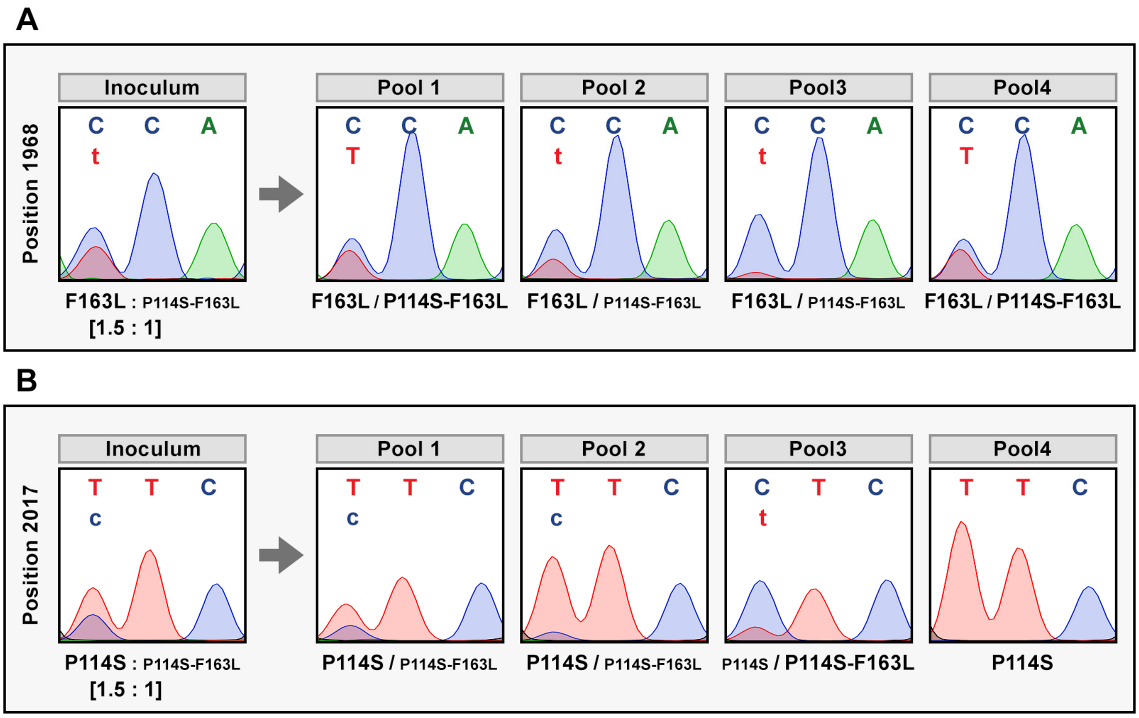

Supplementary Fig. S4: Sequence analysis of viral progeny from *Nicotiana clevelandii* exposed to mixed infections with competing viruses. Eight *N. clevelandii* plants were inoculated by hand-rubbing with DNA mixtures containing the indicated pICPPV-VPgSwCM-R-derived mutant clones. In the two competitions, the single mutant was overrepresented in the inoculum with respect to the double mutant (ratio 1.5:1). Viral progenies were analysed in pools of two plants by reverse transcription-polymerase chain reaction (RT-PCR) and sequencing of a cDNA fragment covering the VPg coding sequence. Images show the chromatograms of VPg codons 114 (position 1968-1970 in the viral genome) **(A)** or 163 (position 2017-2019 in the viral genome) **(B)**. Viruses identified are indicated beneath the chromatograms; smaller letters indicate lower accumulation.

Supplementary Figure S5

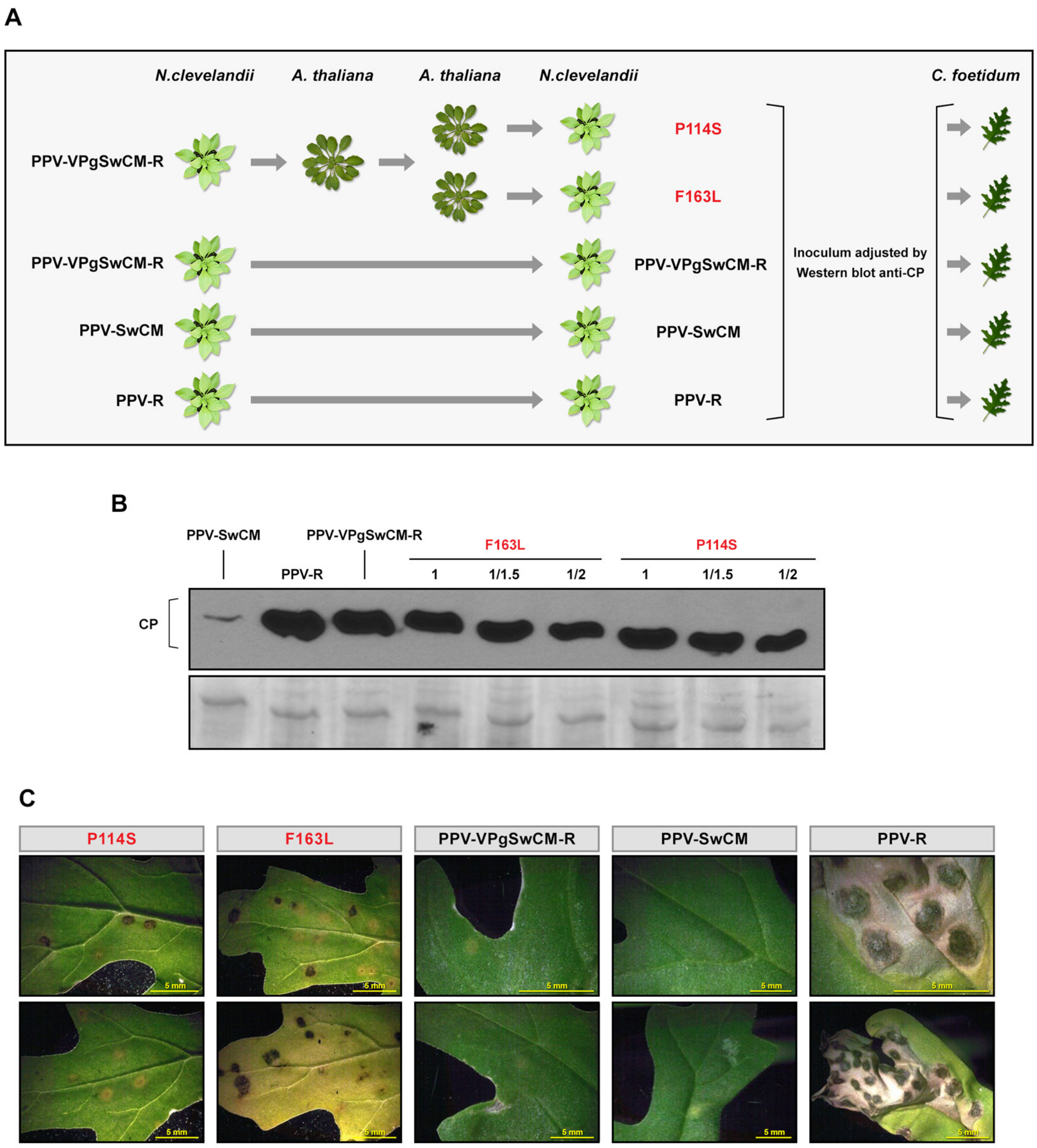

Supplementary Figure S5: Effect of VPg mutations on *Nicotiana clevelandii* infection by *Plum pox virus* (PPV). **(A)** Schematic representation of the experimental approach. **(B)** Prior to *C. foetidum* inoculation, extracts from upper non-inoculated leaves of *N. clevelandii* plants infected with the different viruses, collected at 10 days after inoculation, were subjected to coat protein (CP)-specific immunoblot analysis to estimate virus accumulation, thus allowing to adjust the inoculum doses. Increasing dilutions of the extracts from plants infected with the mutant viruses are indicated. The lower signal observed for the PPV-SwCMp sample is a

consequence of the significantly poorer recognition of PPV-SwCMp CP compared to PPV-R CP by the choice antibody. Blots stained with *Ponceau* red showing the large subunit of the ribulose-1,5-bisphosphate carboxylase-oxygenase (RuBisCO) are included as loading controls in both cases. **(C)** Images of *C. foetidum* leaves taken under visible light at 15 days post infection, after being inoculated with extracts of infected *N. clevelandii* plants. Bar, 5 mm.

Supplementary Figure S6

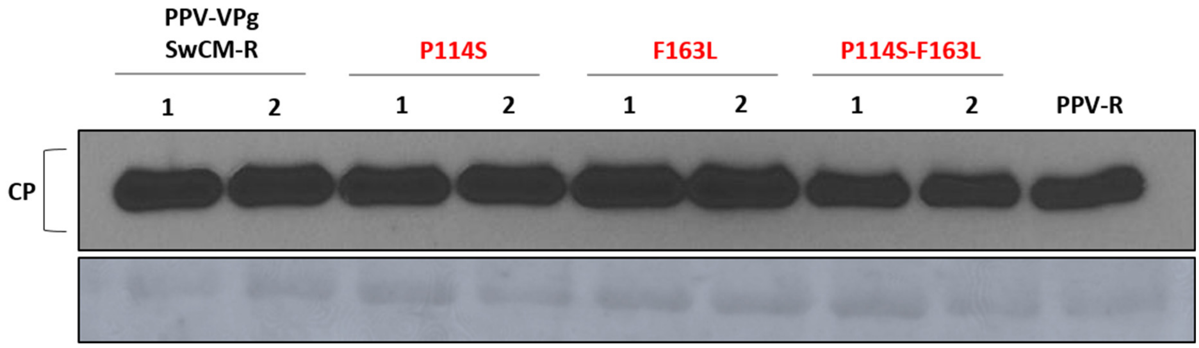

Supplementary Fig. S6: Assessment of viral titers in *Nicotiana clevelandii* extracts employed to inoculate *Chenopodium foetidum* plants. *N. clevelandii* plants were inoculated by hand-rubbing with DNA of the chimeric clone pICPPV-VPgSwCM-R, the indicated chimera-derived mutants (two independent clones, 1 and 2), or the PPV-R clone pICPPV-NK-lGFP. Extracts were prepared from upper non-inoculated leaves collected at 7 days post inoculation and their virus titers were adjusted with extracts from healthy leaves on the basis of a previous quantitative anti-CP immunoblot assay. The adjusted extracts were inoculated by hand-rubbing in *C. foetidum* leaves. Equalization of viral titers in the inocula was verified in the CP-specific immunoblot shown in the figure. Blots stained with *Ponceau* red showing the large subunit of the ribulose-1,5-bisphosphate carboxylase-oxygenase (RuBisCO) are included as loading controls.

Supplementary Figure S7

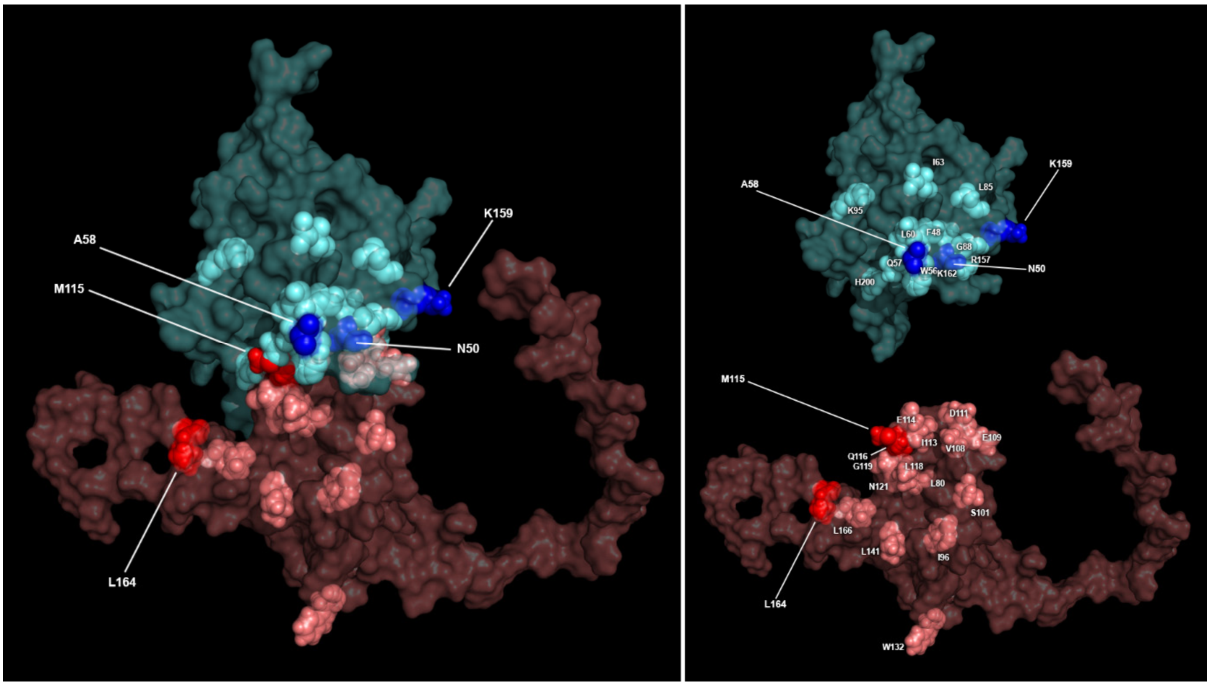

Supplementary Figure S7: HADDOCK-derived structure of the human eIF4E (h-eIF4E) (blue molecule) in complex with the VPg of *Potato virus Y* (PVY) (PVY VPg) (red molecule), obtained by Countinho de Oliveira *et al*. [51], in which the following residues are highlighted: (i) PVY VPg residues perturbed by h-eIF4E binding (in salmon); (ii) residues in PVY VPg equivalent to those of *Plum pox virus* VPg mediating adaptation of the chimeric virus PPV-VPgSwCM-R to *Arabidopsis thaliana* and *Chenopodium foetidum*, P114 [that match one amino acid specified in (i)] and F163 (both in red); (iii) h-eIF4E residues perturbed by PVY VPg binding, whose equivalents are conserved among *A. thaliana*, *Nicotiana clevelandii* and *C. foetidum* (in cyan); and iv) residues in h-eIF4E perturbed by PVY VPg binding, whose equivalents in eIF(iso)4E from *A. thaliana*, *N. clevelandii* and *C. foetidum* are not fully conserved (in bright blue). Structural details were visualized using PyMOL. On the left, h-eIF4E:PVY VPg complex, pointing key residues addressed in this study. On the right, both molecules, separately disposed, indicating all residues reported to be perturbed after protein:protein interaction, according to Counthino de Oliveira *et al*. [51].

Supplementary Table S1

Supplementary Table S1. Primer list

Construct [a]	Primer name	Primer sequence
		Mutators for site directed mutagenesis [b]
P114S	LBR2-F	5´-GGAGATGACAAGATCACA**tCA**CAACACATAATGG-3´
	LBR1-R	5´-GCCATTATGTGTTG**TGa**TGTGATCTTGTCATCTCC-3´
F163L	LBR4-F	5´-GGTTTTCCGGAGAGAGAG**cTC**GAGTTGAGACAAAC-3´
	LBR3-R	5´-GTTTGTCTCAACTC**GAg**CTCTCTCTCCGGAAAACC-3´
		Externals for site directed mutagenesis
P114S<br>F163L<br>P114S-F163L	LBR5-ExF	5´-CTTAAGTTTCGCCAAGCTCGCGATAACCGGAT-3´
	LBR6-ExR	5´-GAATACTGCCATCTCGAGTGCTCACGATGG-3´
		Viral progenies characterization via RT-PCR or IC-RT-PCR [c]
pICPPV-VPgSwCM-R<br>P114S<br>F163L<br>P114S-F163L	2295	5´-CAAAGAAGAAGTTGTTCACCAAGGGTTCAATCGACGGCAGAGAC-3´
	2277	5´-CCTCTGAACAGTGATTTACTTTCATGATCGACTTCCTCACC-3´
pICPPV-NK-lGFP	SM16-F	5´-CTTGGTGGAGGGGCATG-3´
	SM17-R	5´-CTTGCGATTGGATTATAGTCTCTC-3´
		VPg sequencing
SM18-F		5´-GGAGGGGCATGGATG-3´
SM19-R		5´-ATTGGATTATAGTCTCTCAGG-3´
		Cf-eIF(iso)4E gene fragment amplification [d]
SM110-F-deg		5´-AAACAACCMCACAARYTAGAGAG-3´
SM111-R-deg		5´-TCTTTCCACTTCCTWCCAATGCCCATC-3´

[a] Constructs obtained and/or used for the different assays

[b] Mutated codons in bold and underlined. Specific changes are written in lower case

[c] RT-PCR / IC-RT-PCR, Reverse transcription and PCR / preceded by immune-capture

[d] Degenerate primers designed for the identification of the isoform of the eukaryotic translation initiation factor 4E from *Chenopodium foetidum* [Cf-eIF(iso)4E]

Supplementary Table S2

Supplementary Table S2. Effect of specific mutations at the VPg of the *Plum pox virus*, isolate SwCMp (strain C), on the infection of *Chenopodium foetidum*

Inoculum [a]	Total inoculated leaves	Number of lesions [b]	
		9 dpi	15 dpi
PPV-VPgSwCM-R	15	2 (?)	5 + 2 (?)
P114S	21	137	140
F163L	21	353	360
PPV-SwCMp	15	-	-
PPV-R	9	> 60 / leaf<br>3 dead leaves	> 60 / leaf<br>4 dead leaves

[a] Inoculums consisted on leaf extracts of previously infected *Nicotiana clevelandii* plants (according to Supplementary Figure S5), containing the specified viruses. Three leaves per plant were mechanically inoculated by hand-rubbing, at 5 µL extract per leaf.

[b] Total number of lesions, chlorotic or necrotic, counted for the total number of inoculated leaves, or per leaf in the case of the positive control at 9 and 15 days post inoculation (dpi). (?), Doubtful lesions.
