# Supplementary figures and images for: Virus Host Jumping Can Be Boosted by Adaptation to a Bridge Plant Species
Source: Microorganisms. 2021 Apr 11;9(4):805. doi: 10.3390/microorganisms9040805 (PMC8070427; doi:10.3390/microorganisms9040805)

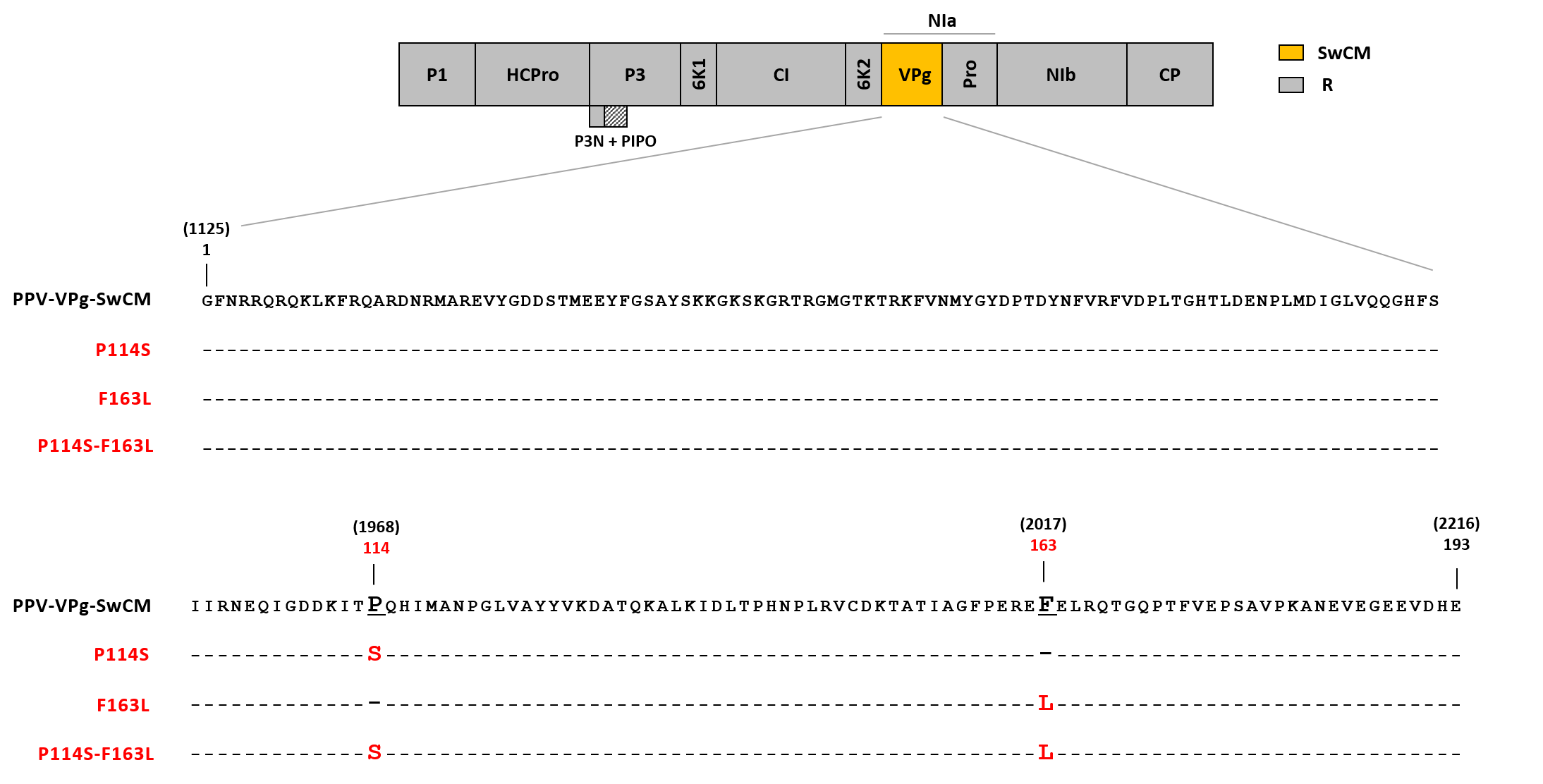

Supplement: Supplementary file 1 [file microorganisms-09-00805-s001.zip › Supplementary Figures_100421_/Supplementary_Figure_S1_.tif]

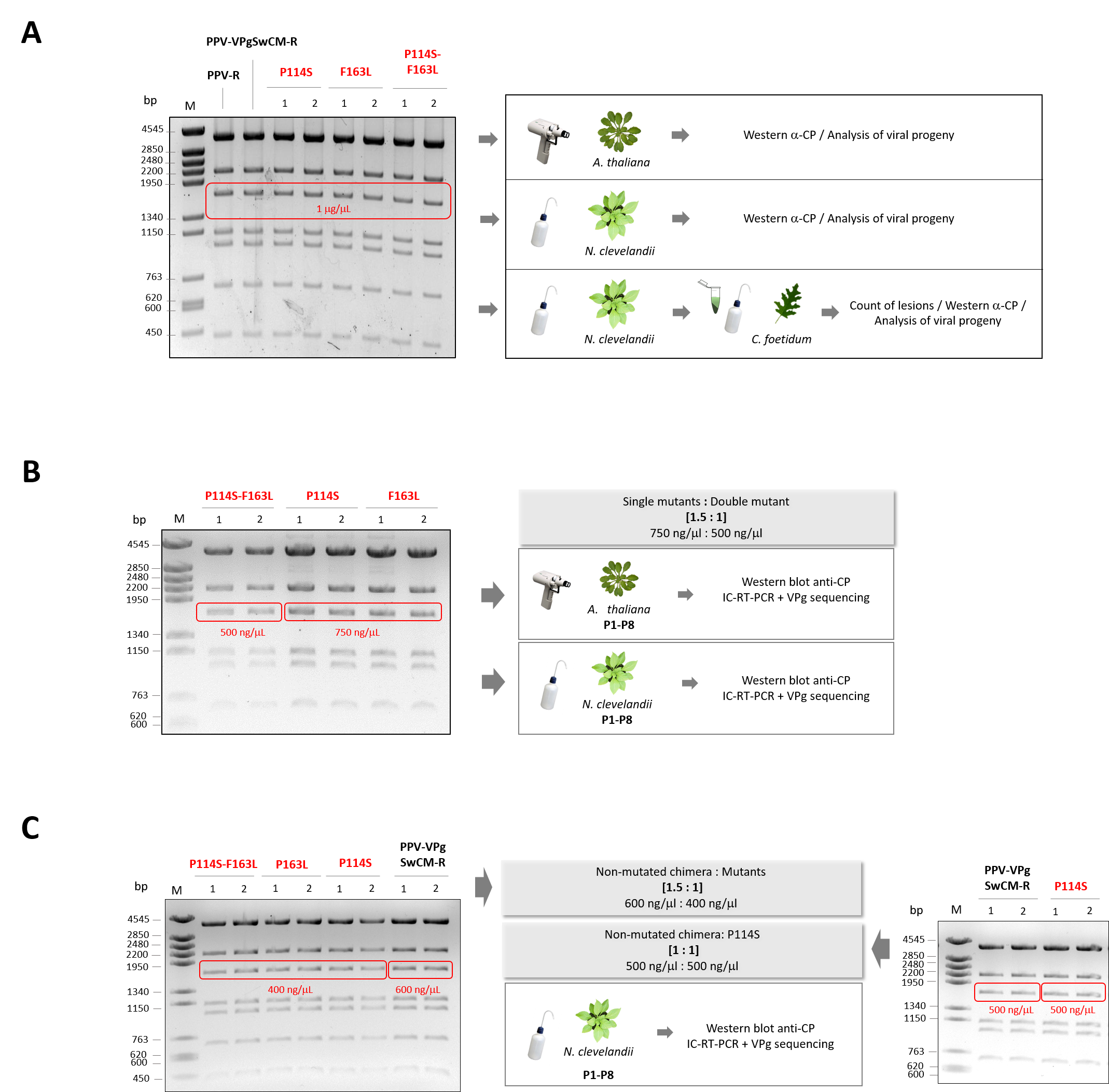

Supplement: Supplementary file 1 [file microorganisms-09-00805-s001.zip › Supplementary Figures_100421_/Supplementary_Figure_S2_.tif]

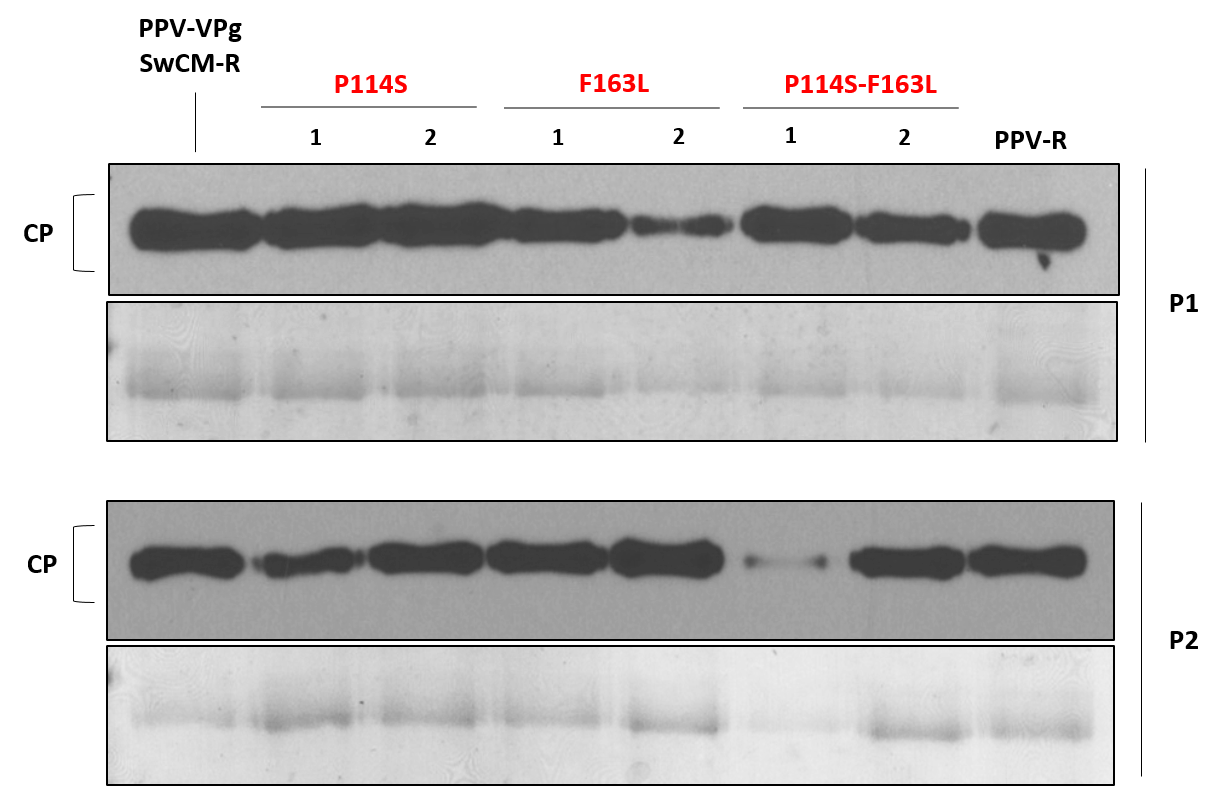

Supplement: Supplementary file 1 [file microorganisms-09-00805-s001.zip › Supplementary Figures_100421_/Supplementary_Figure_S3_.tif]

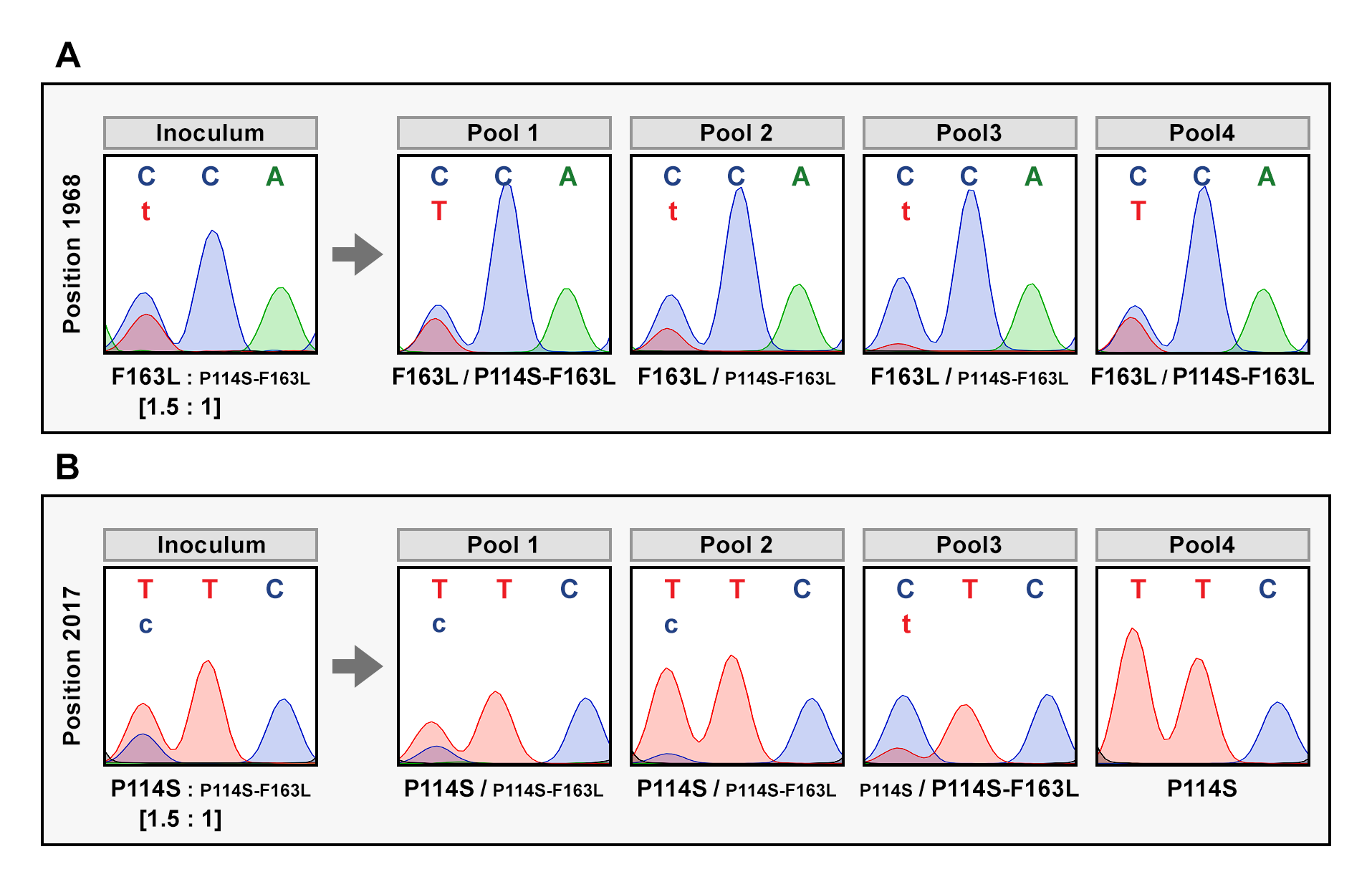

Supplement: Supplementary file 1 [file microorganisms-09-00805-s001.zip › Supplementary Figures_100421_/Supplementary_Figure_S4_.tif]

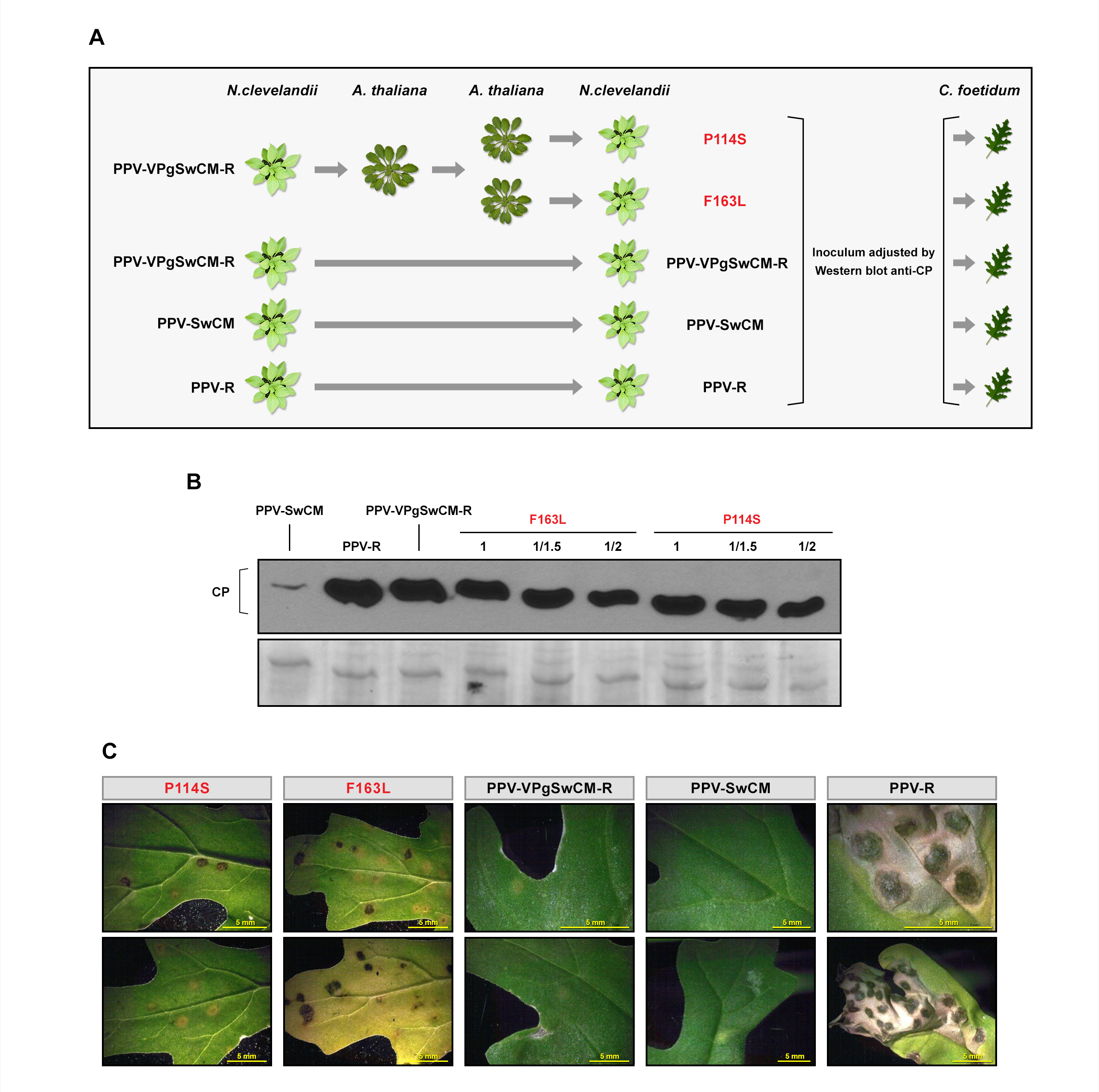

Supplement: Supplementary file 1 [file microorganisms-09-00805-s001.zip › Supplementary Figures_100421_/Supplementary_Figure_S5_.tif]

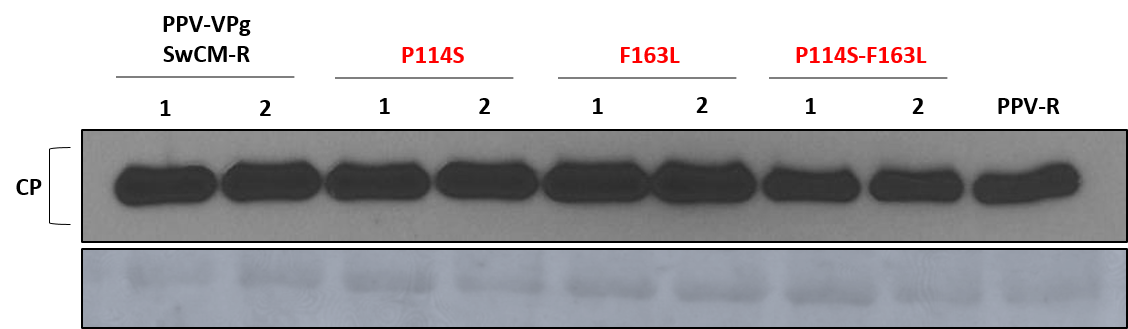

Supplement: Supplementary file 1 [file microorganisms-09-00805-s001.zip › Supplementary Figures_100421_/Supplementary_Figure_S6_.tif]

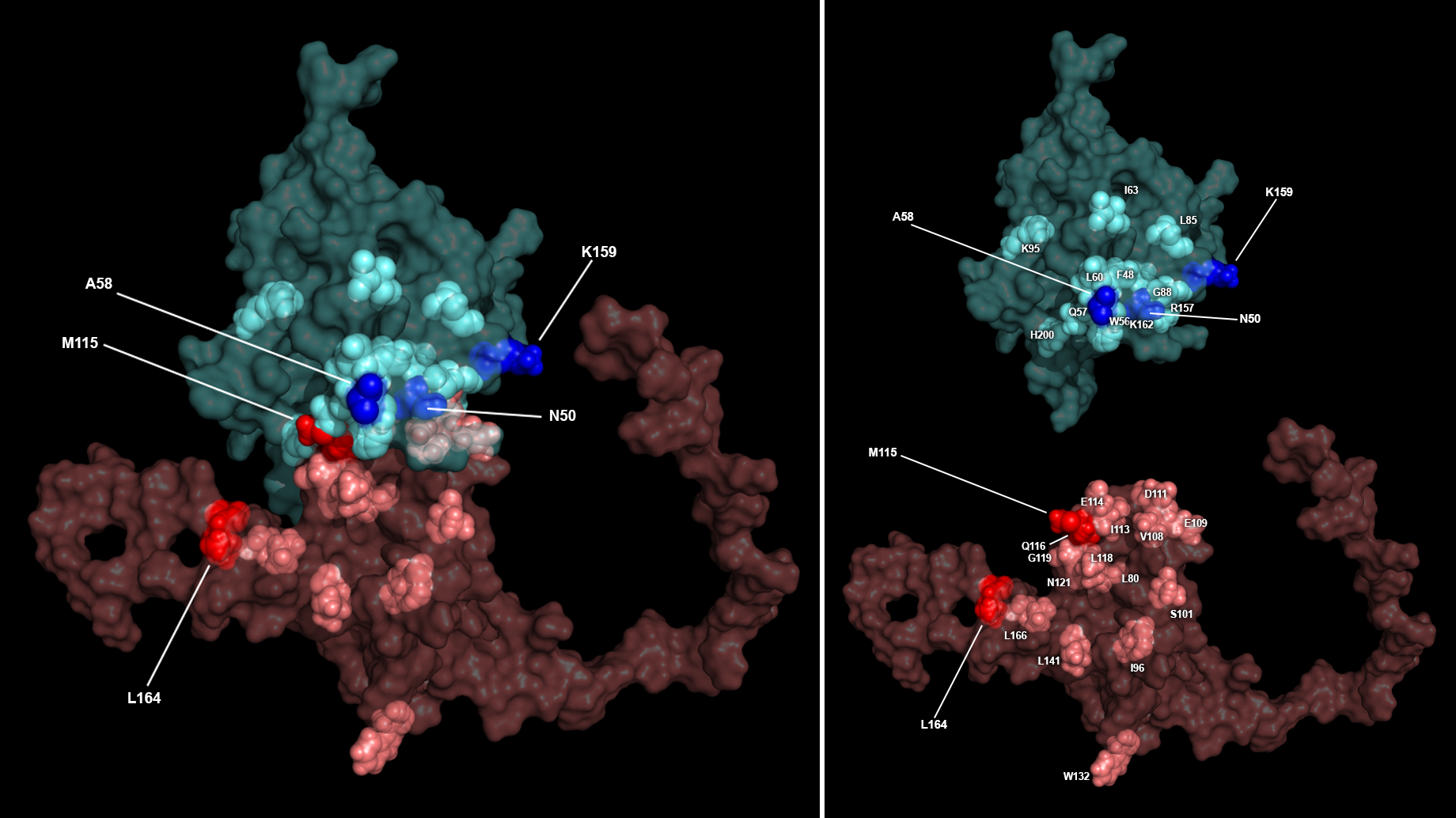

Supplement: Supplementary file 1 [file microorganisms-09-00805-s001.zip › Supplementary Figures_100421_/Supplementary_Figure_S7_.tif]
